# Randomized feasibility trial of directly observed versus unobserved hepatitis C treatment with ledipasvir-sofosbuvir among people who inject drugs

**DOI:** 10.1371/journal.pone.0217471

**Published:** 2019-06-03

**Authors:** Phillip O. Coffin, Glenn-Milo Santos, Emily Behar, Jaclyn Hern, John Walker, Tim Matheson, Elizabeth N. Kinnard, Janelle Silvis, Eric Vittinghoff, Rena Fox, Kimberley Page

**Affiliations:** 1 San Francisco Department of Public Health, San Francisco, California, United States of America; 2 University of California San Francisco, San Francisco, California, United States of America; 3 University of California Berkeley School of Public Health, Berkeley, California, United States of America; Kaohsiung Medical University, TAIWAN

## Abstract

**Aims:**

The advent of direct-acting antivirals for hepatitis C virus (HCV) and limited effectiveness of prevention have generated interest in “Treatment as Prevention” (TasP), in which those most likely to transmit HCV (i.e. people who inject drugs [PWID]) are treated to reduced secondary transmission. However, there are scant data regarding the feasibility of treating PWID at high risk for secondary transmission or the optimal approach to treatment delivery.

**Methods:**

We conducted a 2:1 randomized trial of modified directly-observed (mDOT) versus unobserved HCV treatment with ledipasvir-sofosbuvir daily for 8 weeks among PWID with 36 weeks of follow-up in San Francisco from 2015–2017. We evaluated recruitment-enrollment, treatment completion, end-of-treatment and 12-week response, and reinfection rate.

**Results:**

Of 83 individuals eligible for screening, 72 (87.6%) attended the screening visit, 33 were eligible, and 31 enrolled; mean age was 42 years, 81% were male, 74% white. All but one participant (in the mDOT arm) completed treatment and 89.4% of mDOT and 96.6% of unobserved arm visits were attended. HCV was undetectable for 96.8% (30/31) at end of treatment and 89.7% (26/29) 12 weeks later (1 relapse, 1 reinfection), with no differences by arm. Two additional reinfections were subsequently identified, for a reinfection rate of 16.3 (95% CI 5.3–50.5) per 100 person-years of observation.

**Conclusions:**

It was feasible to recruit active PWID for HCV treatment and achieve high retention, viral response, and satisfaction with either mDOT or unobserved protocols, supporting treatment of PWID at risk of transmitting HCV to others. The reinfection rate suggests we successfully reached a high-risk population and that successful HCV TasP initiatives may aim to be sufficient in scope to significantly lower prevalence in the community.

**Trial registration:**

clinicaltrials.gov NCT02609893.

## Introduction

One percent of the world’s population (71.1 million people) are believed to be viremic with hepatitis C virus (HCV).[1] In the United States, HCV-related mortality surpasses 60 other nationally notifiable infectious conditions combined.[2–4] HCV primarily affects people who inject drugs (PWIDs), 43% of whom are believed to be infected.[2,5] Moreover, the current resurgence of HCV in the U.S. is related to increases in injection drug use.[2,3] Once HCV is introduced into a network of PWIDs, it has been shown to circulate rapidly through injection equipment.[3,6,7]

The World Health Organization and U.S. Centers for Disease Control and Prevention have both set forth goals of reducing HCV incidence by 90% by 2030.[8,9] Though critically important harm reduction interventions, syringe access programs (SAPs) alone have not proven sufficiently effective in reducing HCV incidence.[10] The most optimistic findings suggest that decades of SAPs have delayed HCV infection such that most PWIDs do not become infected until they have been injecting drugs for about two years, whereas the median duration of injecting drugs is 10 years.[11,12] In contrast, mathematical models suggest that treating PWIDs for HCV could substantially reduce incident infections.[13–17]

Referred to as “treatment as prevention” (TasP), this approach, whereby treatment is used to decrease the likelihood of forward transmission, has been widely employed for HIV. For HCV, treatment is curative and is expected to have a similar effect on transmission as that seen for HIV.[18,19] TasP for HCV appears feasible with novel direct acting antivirals (DAAs), yet concerns about costs of DAA therapy and subsequent reinfections among PWID persist, limiting payer coverage and provider willingness to treat PWIDs.[14,18,20]

We sought to evaluate the feasibility and acceptability of HCV TasP among PWIDs by assessing enrollment, retention, medication adherence, treatment completion, treatment success, and reinfection rates in two models of delivering HCV treatment to people who were actively injecting drugs. We focused on those who were actively injecting with other people present to ensure we were reaching people who could be at risk for transmitting HCV to others.

We hypothesized that we would achieve high rates of enrollment, retention, treatment completion, and viral suppression, without observable differences by study arm.

## Methods

To test the feasibility and acceptability of treating actively-injecting PWID for HCV and compare methods of treatment delivery, we conducted a two-arm randomized trial (the BYE-C trial) of modified directly-observed treatment (mDOT) versus unobserved treatment with 8 weeks of ledipasvir-sofosbuvir 80mg/400mg oral once daily fixed-dose combination (LDV-SOF) among 31 PWID at the San Francisco Department of Public Health from 2015–2017 (S1 File: Study Protocol). Study procedures were approved by the University of California, San Francisco, Institutional Review Board (IRB#15–16278).

### Recruitment

Potential participants were recruited from the UFO Study of HCV incidence among PWID under 30 years of age[21] and by word-of-mouth from syringe access programs and other healthcare settings. Interested individuals were administered a questionnaire by phone to establish preliminary eligibility and schedule an in-person screening. At the screening visit, participants gave informed consent and were evaluated for eligibility using the following inclusion criteria: ≥18 years old; 2 consecutive positive HCV RNA tests at least 6 months apart; HCV genotype 1; HCV RNA <6 million copies by Roche TaqMan^®^ assay; drug injection in past 30 days, with others present in past 12 months, and track marks on exam; able to speak English; no plans to leave the San Francisco Bay Area in ensuing 9 months; and, for women of childbearing age, a negative pregnancy test, not actively nursing, and agreement to use birth control during treatment.

Exclusion criteria included prior hepatic decompensation or evidence of hepatic cirrhosis by either Fib4≥3.25 or fibrosis-cirrhosis index≥1.25; chronic liver disease for non-HCV reason (e.g., Wilson’s disease, alpha-1-antitrypsin deficiency, cholangitis) except iron overload; HIV seropositive; HBV surface antigen positive; previously treated for HCV; use of medications that affect LDV-SOF pharmacokinetics (e.g., proton pump inhibitors, anticonvulsants, rifamycins, rosuvastatin, herbs [e.g., St. John’s wort, silymarin, echinacea]; amiodarone); current gastrointestinal disorder that could affect LDV-SOF absorption; prior solid organ or bone marrow transplantation; active cancer treatment; known hypersensitivity to LDV-SOF or formulation excipients; any other conditions precluding participation as determined by the medical director.

### Procedures

Two screening visits assessing for eligibility included physical exam; medical history; blood count with differential, comprehensive metabolic panel, coagulation studies, hepatitis B surface antigen, HIV antigen/antibody, HCV RNA and genotype (or recent lab documentation), urine beta-HCG (for women), qualitative urine testing for drug metabolites using the Reditest 11 Panel-Dip (Redwood Toxicology Laboratory, Santa Rosa, CA), and stored blood sample for potential HCV sequencing to distinguish recurrence from reinfection. Enrollment occurred within 30 days of screening and included urine drug screen, beta-HCV (for women), vital signs, randomization to study arm, motivational interviewing–based counseling for injection risk reduction and medication adherence, administration of the Audio Computer Assisted Self Interview (ACASI), and first DOT dosing (mDOT arm) or medication dispensation (unobserved dosing). Participants were randomized 2:1 to mDOT or unobserved dosing using permuted blocks of randomly selected sizes 3 and 6 generated using a STATA program (College Station, Texas). mDOT participants attended the clinic daily Monday-Friday for mDOT dosing and received LDV-SOF in a Wisepill dispenser (Wisepill Technologies; Somerset West, South Africa) each weekend; unobserved participants received 7 tablets weekly of LDV-SOF in a Wisepill dispenser at each weekly visit.

All participants attended weekly visits including mDOT dosing or medication dispensation, study drug dosing compliance (pill count), review of concomitant medications and adverse events, and urine drug screen. Week 2 also included blood count, comprehensive metabolic panel, and HCV RNA. Week 4 also included symptom-driven physical examination, vital signs, ACASI, HCV RNA, and motivational interviewing for medication adherence. Week 8 also included symptom-driven physical examination, vital signs, blood count with differential, comprehensive metabolic panel, HCV RNA, HIV antibody/antigen, ACASI, and a qualitative interview.

Participants were seen 1 week post-treatment for review of adverse events, provision of HCV RNA results, and motivational interviewing–based risk reduction counseling; and again at post-treatment weeks 12 and 36 for urine drug screen, ACASI, HCV RNA, stored samples for HCV sequencing, and, for week 12, motivational interviewing-based risk reduction counseling.

Participants were compensated $15 per screening visit; $20 for enrollment; $20 for weekly visits with an additional $5 for weeks 2, 4, and 8; $15 for post-treatment week 1; and $50 each for post-treatment weeks 12 and 36. At each weekly visit, mDOT dosing participants received a 10-trip transit card and unobserved participants received a two-trip transit card to assist with round-trip transportation to the study site.

### Measures

We assessed the feasibility of treating active PWIDs for HCV with LDV-SOF by mDOT versus unobserved dosing by calculating the following measures: 1) proportion eligible among those screened; 2) proportion enrolled among those eligible; and 3) study retention and treatment completion rates, overall and by arm.

In addition, we calculated the end-of-treatment and 12-week post-treatment HCV viral status (EoT and SVR-12, respectively), and reinfection rate, overall and by arm. Consistent with other studies, for reinfection cases, we estimated the date of HCV reinfection by using the midpoint date between the date of the last undetectable HCV RNA test and the date of the first detectable HCV RNA test.[22] Time zero for the reinfection rate analyses was defined as the date of end of treatment. For participants without reinfection, person-time was calculated by taking the difference between the date of the last undetectable HCV RNA test, and time zero. A Kaplan-Meier curve was used to describe time to HCV reinfection among participants who achieved HCV clearance at end of treatment (n = 30).

We evaluated the acceptability of mDOT versus unobserved dosing, the percent of treatment medication adherence to LDV-SOF, as measured by the percent of doses taken, which was assessed using mDOT doses and weekend WisePill data for the mDOT arm, and WisePill data for the unobserved dosing arm. Pill counts were used as another measure of adherence.

ACASI was completed at enrollment, weeks 4, 8, and post-treatment weeks 12 and 36. Questions assessed injection drug use and injection risk behavior; partner injection practices, substance use, and HCV status; quality of life (Short Form Survey-12 [SF-12]); depression (Center for Epidemiologic Studies Depression Rating Scale[23]); drug dependence (Severity of Dependence Scale[24]); and participant satisfaction with treatment (week 8 only).

### Data analysis

Between-group differences in retention and completion were assessed using Fisher’s exact tests for categorical data and Wilcoxon ranksum tests for continuous data. We compared the proportion of participants with EoT response and SVR-12 between arms using Fisher’s exact tests. Between-arm differences for adherence during treatment were assessed using Wilcoxon ranksum tests. Differences in adverse events were assessed using Fisher’s exact tests. For all the between group comparisons above, we used 2-sided tests and an α cut-off of 0.05 to determine statistical significance. All analyses were conducted in STATA version 15 (College Station, TX).

## Results

### Subjects

**Fig 1:**
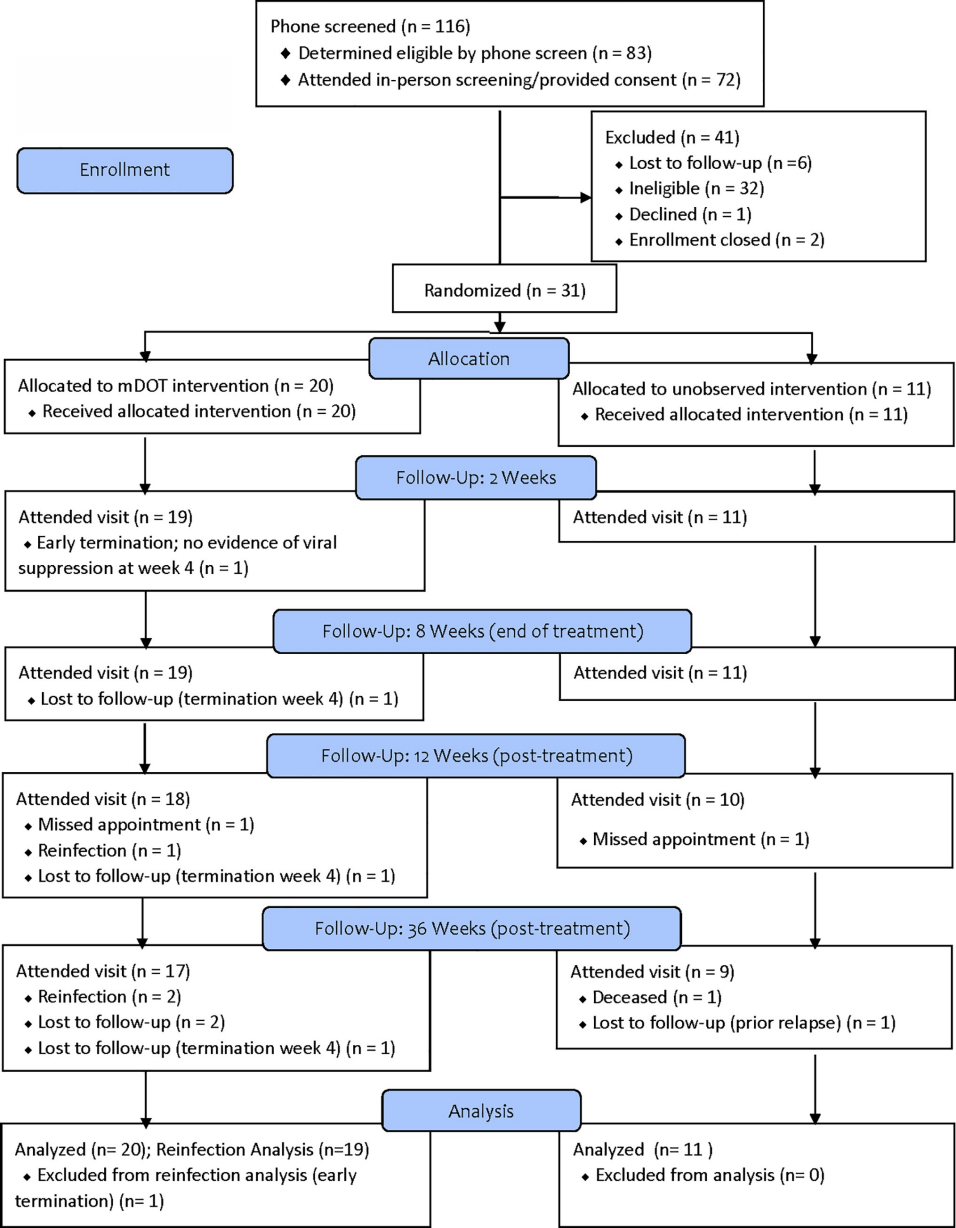
BYE-C consort diagram.

Of 116 individuals prescreened, 83 were eligible for screening, 72 (87.6%) attended the two screening visits and provided consent, and 33 were eligible for the trial (**Fig 1**). The most common reasons for being ineligible included lab results (n = 16) and not having HCV genotype 1 (n = 10). Thirty-one participants enrolled and were randomized (DOT arm n = 20; unobserved arm = 11); mean age was 42.4 years, 80.7% were male, and 74.2% were white. Almost 50% reported not being housed for most of the past 30 days, although 77.4% had a regular healthcare provider. Almost half (45.2%) reported daily injection over the past 30 days, with 77.4% reporting heroin injection during that period. Participants had a mean of 6.2 injection partners in the past 30 days. Almost half of participants (45.2%) reported using a syringe to backload or piggyback in the past 30 days and 35.5% used the same cooker at the same time as someone else. The only significant differences by study arm were that participants in the mDOT arm were more likely to have used the same syringe before someone else used it in the past 30 days, and had significantly lower physical health scores in SF-12 (**Table 1, S2 File: Dataset**).

**Table 1:** Baseline characteristics by randomization arm (N = 31).

	Unobserved (n = 11)		Modified DOT (n = 20)		Total (N = 31)		P-value
	N	(%)	N	(%)	N	(%)	
**Age, mean (SD)**	39.10	(12.0)	44.10	(11.8)	42.43	(11.9)	0.29
**Gender**							
Female	2	(18.2)	4	(20.0)	6	(19.4)	>0.99
Male	9	(81.8)	16	(80.0)	25	(80.7)	
**Race/Ethnicity**							
White	8	(72.7)	15	(75.0)	23	(74.2)	>0.99
Non-white	3	(27.3)	5	(25.0)	8	(25.8)	
**Employment Status**							
Unemployed	9	(81.8)	15	(75.0)	24	(77.4)	0.83
Employed	1	(9.1)	4	(20.0)	5	(16.1)	
Did not report	1	(9.1)	1	(5.0)	2	(6.5)	
**Annual Income**							
No income	6	(54.6)	8	(40.0)	14	(45.2)	0.93
$1 - $9,999	2	(18.2)	5	(25.0)	7	(22.6)	
$10,000 and above	3	(27.3)	6	(30.0)	9	(29.0)	
**Housing Status (past 30 days)**							
Housed	4	(36.4)	12	(60.0)	16	(51.6)	0.27
Not Housed	7	(63.6)	8	(40.0)	15	(48.4)	
**Education**							
Less than high school	3	(27.3)	4	(20.0)	7	(22.6)	0.24
High school graduate	2	(18.2)	10	(50.0)	12	(38.7)	
Some college or more	5	(45.5)	6	(30.0)	11	(35.5)	
**Has a Regular Health Care Provider**							
No	3	(27.3)	4	(20.0)	7	(22.6)	0.68
Yes	8	(72.7)	16	(80.0)	24	(77.4)	
**Health Status in SF-12**							
General Health Score, mean (SD)	43.0	(10.5)	39.3	(11.3)	40.6	(11.0)	0.07
Physical Health Score, mean (SD)	43.6	(7.3)	37.8	(6.4)	40.0	(7.2)	0.02
Mental Health Score, mean (SD)	30.3	(8.2)	34.0	(8.5)	32.6	(8.4)	0.10
**Daily Injection Drug Use, Past 30 Days**	4	(36.4)	10	(50.0)	14	(45.2)	0.66
**Number of Injection Partners, Past 30 Days, mean (SD)**	9.0	(23.6)	4.6	(5.1)	6.2	(14.6)	0.61
**Substances Injected, Past 30 Days**							
Heroin	9	(81.8)	15	(75.0)	24	(77.4)	>0.99
Prescription Opioids	4	(36.4)	5	(25.0)	9	(29.0)	0.80
Cocaine/Crack	4	(36.4)	3	(15.0)	7	(22.6)	0.47
Methamphetamine	8	(72.7)	13	(65.0)	21	(67.7)	>0.99
**Injection Risk Behaviors, Past 30 Days**							
Used same syringe after someone else used it	2	(18.2)	3	(15.0)	5	(16.1)	0.57
Used same syringe before someone else used it	1	(9.1)	8	(40.0)	9	(29.0)	0.03*
Used same cooker at same time as someone else	3	(27.3)	8	(40.0)	11	(35.5)	0.86
Used same cooker after someone else used it	3	(27.3)	4	(20.0)	7	(22.6)	0.85
Used same cooker before someone else used it	3	(27.3)	4	(20.0)	7	(22.6)	0.85
Used same cotton after someone else used it	2	(18.2)	7	(35.0)	9	(29.0)	0.25
Used same cotton before someone else used it	2	(18.2)	5	(25.0)	7	(22.6)	0.55
Used syringe to backload or piggyback	6	(54.6)	8	(40.0)	14	(45.2)	0.81

### Study retention and adherence

During the treatment phase, participants in the unobserved arm attended 96.6% of weekly visits while participants in the mDOT arm attended 96.3% of weekly visits (p = 0.89). In addition, participants in the mDOT arm attended 89.4% of daily dosing visits; 91.6% of daily visits were completed after excluding one participant in the mDOT arm who returned only at week 4 after enrollment with no evidence of HCV viral suppression and thus was discontinued early. All other participants completed treatment (96.8%).

Adherence to unobserved doses was measured using WisePill dispensers and by pill counts. Mean adherence by WisePill dispensers was 39.2% (SD: 36.8) in the unobserved arm and 49.9% (SD: 38.6) for weekend doses in the mDOT arm (P = 0.66). Only 36.4% of participants in the unobserved arm and 60.0% of participants in the mDOT arm demonstrated > 50% adherence by WisePill (P = 0.27). However, several participants never opened their WisePill dispensers and the dispenser was not returned in 53% of dispensing visits, with no difference by arm (P = 0.72), resulting in use of alternative pill containers for those weeks. Among visits that involved a returned WisePill dispenser or alternative pill containers, adherence by pill count was 99.5%, again with no difference by arm (P = 0.70).

### Viral response and reinfection

HCV RNA was undetectable for 29/31 (93.5%, 95% exact confidence interval (exCI) 78.69% to 99.2%) at 2 weeks and 30/31 (96.8%, 95% exCI 83.3% to 99.9%) at EoT (DOT 95%, unobserved 100%; P>0.99). One participant in the mDOT arm was terminated early due to not attending visits after enrollment until week 4.

Post-treatment week 12 was attended by 28 participants, with one viral relapse based on HCV sequencing and one reinfection based on genotype Including the participant terminated early, we can confirm SVR-12 for 26/29 participants (89.7%, 95% exCI 72.6% to 97.8%). Two additional participants who missed the 12-week visit were HCV RNA-negative at the 36-week visit, suggesting an SVR-12 for the full sample of 28/31 (90.3%, 95% exCI 74.2% to 98.0%).

**Fig 2:**
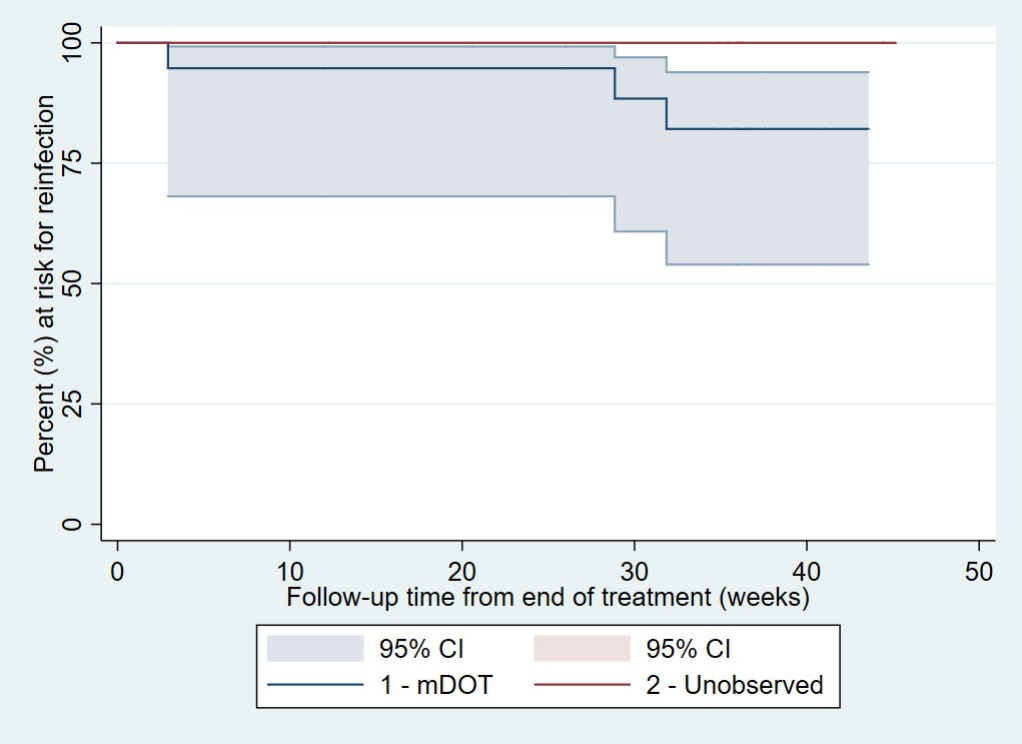
Proportion at risk for reinfection among PWID who achieved HCV clearance at end of treatment (n = 30). *Excludes one participant who was terminated early (mDOT arm); 20 participants at risk at start of mDOT and 10 participants at risk throughout Unobserved.

Post-treatment week 36 was attended by 26 participants Of the five participants who missed the visit, one was deceased, two were known to be HCV RNA-positive from prior visits, and two were HCV RNA-negative at 12 weeks. We identified 2 additional reinfections based on genotype, and are thus able to confirm absence of HCV RNA for 23/26 participants at the week 36 visit and infer an SVR-36 in 23/28 participants (82.1%, 95% exCI 63.1% to 93.9%). A search of local healthcare system records was unable to identify additional clinical laboratory values to enhance these data (Table 2 and Fig 2).

**Table 2:** BYE-C visit completion and hepatitis C viral outcomes.

	Mean Weekly Visit Completion (through week 8)		End of treatment response (through week 8)		SVR-12 (through post-treatment week 12)		Re-infection rate (through post-treatment week 36)	
	Mean %	[SD]	%	[95% exCI]	%	[95% exCI]	N*	[95% CI]
Unobserved	96.6	[18.3]	100	[71.5–100]	90	[55.5–99.7]	0	N/A
Modified DOT	96.3	[19.1]	95	[75.1–99.8]	89.5	[66.9–98.7]	25.9	[8.3–80.2]
Overall	96.4	[18.7]	96.8	[83.3–99.9]	89.7	[72.6–97.8]	16.3	[5.3–50.5]
P-value	p = 0.89^a^		p > 0.99^b^		p > 0.99^b^		p = 0.13^c^	

Notes:

N* = number of reinfections per 100 person years. For this study, person-time represents an estimate of the actual time-at-risk for reinfection, in years, that all participants contributed to this study since end of treatment (see methods section for more details).

a = based on Wilcoxon ranksum test

b = based on Fisher’s exact test

c = based on Maximum likelihood estimate of rate ratio

The participant, in the unobserved arm, who relapsed at week 12 after EoT response had HCV genotype 1a/1b with RNA of 1.3 million IU/mL at baseline, 240 IU/mL at 2 weeks of treatment, and 840,000 IU/mL 12 weeks after treatment. That participant attended all weekly visits, although WisePill was lost after two weeks of treatment, limiting the ability to confirm medication adherence.

We observed a reinfection rate of 16.3 (95% CI 5.3–50.5) per 100 person-years overall during the 18.42 years of total analysis time at risk and under observation for the study. The reinfection rate for the mDOT arm was 25.9 (95% CI 8.3–80.2) per 100 person-years. There were no reinfections after 8.5 person-years of follow-up in the unobserved arm.

### Safety

There were no significant differences in frequency of adverse events between arms (P>0.99). There were three serious adverse events during this study, none of which were deemed related to study drug. One participant in the mDOT arm experienced bacterial endocarditis with subsequent mitral valve replacement. One participant in the unobserved arm was hospitalized for bacteremia, epidural abscesses, and subsequent spinal surgeries. Finally, one participant in the unobserved arm was struck and killed by a train. The most common adverse events were: fatigue or malaise (overall = 8/31 (25.8%), mDOT = 4/20 (20.0%), unobserved = 4/11 (36.4%); P = 0.41); high non-fasting glucose (overall = 5/31 (16.1%), mDOT = 3/20 (15.0%); unobserved = 2/11 (18.2%); P>0.99); hypertension (age 18+ years) (overall = 5/31 (16.1%), mDOT = 2/20 (10.0%); unobserved = 3/11 (27.3%); P = 0.32); psychiatric disorders (overall = 4/31 (12.9%), mDOT = 2/20 (10.0%); unobserved = 2/11 (18.2%); P = 0.60); and nausea (overall = 3/31 (9.7%), mDOT = 1/20 (5.0%); unobserved = 2/11 (18.2%); P = 0.28). No participants discontinued study drug due to adverse events.

### Participant satisfaction

At EoT (week 8), all 29 participants who completed the questionnaire reported that the services received helped them deal more effectively with their problems, with 26 (89.7%) reporting they helped a great deal, and no significant differences by study arm. Additionally, 28 (97%) of participants reported receiving “excellent” quality of service during the study, 27 (93%) reported “definitely” getting the kind of service they wanted, 25 (86%) reported having all of their needs met, 28 (97%) reported that they would “definitely” recommend the study to a friend need of similar help, 26 (90%) reported being “very satisfied” with the amount of help they received, and 27 (93%) reported that they were “very satisfied” with the help they received in a general sense. For all these constructs, there were no difference between arms.

## Discussion

HCV treatment for PWID, when available, is often restricted to controlled settings such as methadone maintenance treatment programs. These settings provide an important service, however may not reach those PWID most at risk for transmitting HCV to others, and thus may not achieve the aims of TasP. We found that community-recruited PWID who were actively injecting drugs with others present, and thus at high risk for secondary transmission, could be successfully recruited, treated, and retained in DAA treatment. Moreover, we observed no differences between mDOT and unobserved dosing. Although this pilot study was underpowered to confirm equivalence between the two arms, results raise the prospect of lower threshold approaches to treating HCV among this population. Much like tuberculosis treatment, a flexible model ranging from mDOT to unobserved treatment may be the most appropriate.

While we anticipated high levels of enthusiasm for treating HCV infection at the time of this study, only seven study participants were recruited from the UFO study of young PWID, initially our only planned recruitment site, in which cumulative HCV incidence was 26.7 per 100 person-years.[21] As this population is at high risk of acquiring and transmitting HCV, they are extremely important to enroll in an HCV TasP intervention.[25–27] This relatively low interest has been explored in an analysis by UFO study staff, who note that other priorities may supersede HCV treatment [28], and suggests that additional efforts (e.g., locating services at a trusted drop-in center or conducting a public campaign) would be needed to reach this population successfully.

Adherence to mDOT dosing was good, although our measure of adherence to unobserved doses was unreliable, with estimates ranging from 39.2% from WisePill data to 99.5% from pill counts. The adherence to mDOT dosing and visits, as well as viral response rates, suggest that this population, even with high rates of homelessness, can be treated successfully outside of more regulated settings. The repeated loss of WisePill dispensers, which limited our ability to measure adherence to unobserved dosing, demonstrates the instability in the lives of people who most need to be treated for TasP to be successful. It is likely that adherence was less than in other clinical trials [29–31], yet these new, highly effective DAAs have been shown in previous literature to be successful with suboptimal adherence.[32] Thus, our study both adds to the growing evidence alleviating concerns about optimal adherence being necessary to achieve SVR, and suggests that any effort to expand HCV TasP should incorporate multiple modes of medication delivery. Having medication available at low-threshold drop-in centers, having ways to store medications for those who do not have a safe place, and allowing people to take medications with them when appropriate, are all important to ensure accessibility and breadth of any TasP initiative.

Reinfection remains a concern for those considering treating PWID for HCV. While reinfection rates were low (4–5%) in the era of interferon,[33] our results raise the possibility of higher rates for PWID who continue to inject in the era of DAAs. The confidence interval for reinfection in this pilot study is wide, limiting our ability to make firm conclusions, however it is possible that we identified and treated a particularly high risk cohort of PWID, as injecting with others present was required for enrollment, and/or that the relative ease of DAA treatment reduces the incentive to avoid reinfection with HCV. Although all reinfections occurred in the mDOT dosing arm, we are unable to identify any mechanism to suggest that reinfection was a result of study arm. In addition to education regarding safer injection, including consideration of drug sharing/splitting practices,[34] HCV TasP initiatives may need to be large enough to meaningfully lower prevalence in order to further reduce the rate of reinfection.

There are a number of limitations to this study. First, this was a pilot study, and thus confidence intervals are wide and we are unable to confirm the equivalence of study arms. Second, participants were from the San Francisco Bay Area, and predominantly male and white, which may limit the generalizability of our findings. Third, repeated loss of medication adherence monitoring devices precluded us from establishing if a specific threshold of adherence was necessary to achieve SVR, highlighting the challenges of collecting reliable medication adherence data among PWID. Future studies should endeavor to evaluate DAA adherence using other methods that are not dependent on the use of monitoring devices or pill counts, such as body fluid samples.

In summary, our pilot trial suggests that PWID at risk of transmitting HCV to others might be successfully treated with both mDOT and unobserved dosing protocols. Successful treatment as prevention for HCV may need to incorporate innovative efforts to reach PWID who have competing priorities for time, provide varied and flexible approaches to medication delivery in convenient locations, and be of sufficient scope to meaningfully lower prevalence.

## Supporting information

S1 File San Francisco department of public health clinical trial protocol: Pilot treatment as prevention for HCV among persons who actively inject drugs. (DOCX)

S2 File BYE-C dataset. (CSV)

S3 File Consort 2010 checklist (1).pdf. (PDF)
