# Supplementary material for: Randomized feasibility trial of directly observed versus unobserved hepatitis C treatment with ledipasvir-sofosbuvir among people who inject drugs
Source: PLoS One. 2019 Jun 3;14(6):e0217471. doi: 10.1371/journal.pone.0217471 (PMC6546233; doi:10.1371/journal.pone.0217471)
Supplement: S1 File — (DOCX) [file pone.0217471.s001.docx]

san francisco department of public health

clinical study protocol

| Study Title: | Pilot Treatment as Prevention for HCV Among Persons Who Actively Inject Drugs |
| --- | --- |
|  |  |
| Sponsor: | San Francisco Department of Public Health  25 Van Ness Avenue, Suite 500  San Francisco, California 94102 |
|  |  |
| IND No.: | N/A |
|  |  |
| Indication: | Hepatitis C Virus Infection |
|  |  |
|  |  |
| Study Director: | Name: Phillip O. Coffin, MD MIA Telephone: 415-437-6282 E‑mail: [Phillip.coffin@sfdph.org](mailto:Phillip.coffin@sfdph.org) |
|  |  |
| Protocol Version/Date: | Original: 25 September 2015  Amendment 1: 20 October 2015 Amendment 2: 22 October 2015  Amendment 3: 27 June 2017  Amendment 4: 23 April 2019 (corrected name of quality of life measure) |

TABLE OF CONTENTS

[TABLE OF CONTENTS 2](#_Toc415569523)

[LIST OF IN-TEXT TABLES 4](#_Toc415569524)

[PROTOCOL SYNOPSIS 5](#_Toc415569525)

[GLOSSARY OF ABBREVIATIONS AND DEFINITION OF TERMS 14](#_Toc415569526)

[1. INTRODUCTION 16](#_Toc415569527)

[1.1. Background 16](#_Toc415569528)

[1.2. Ledipasvir/Sofosbuvir Fixed-Dose Combination 16](#_Toc415569529)

[1.3. Rationale for the Current Study 16](#_Toc415569530)

[1.4. Rationale for Dose Selection of Ledipasvir/Sofosbuvir Fixed Dose Combination 17](#_Toc415569531)

[1.5. Overall Risk/Benefit Assessment 17](#_Toc415569532)

[1.6. Compliance 17](#_Toc415569533)

[2. OBJECTIVES 18](#_Toc415569534)

[3. STUDY DESIGN 19](#_Toc415569535)

[3.1. Study Treatment and Duration of Treatment 19](#_Toc415569536)

[3.2. Treatment Discontinuation Criteria 19](#_Toc415569538)

[4. SUBJECT POPULATION 20](#_Toc415569539)

[4.1. Number of Subjects and Subject Selection 20](#_Toc415569540)

[4.2. Inclusion Criteria 20](#_Toc415569541)

[4.3. Exclusion Criteria 20](#_Toc415569542)

[5. INVESTIGATIONAL MEDICINAL PRODUCTS 22](#_Toc415569543)

[5.1. Description and Handling of LDV/SOF 22](#_Toc415569544)

[5.1.1. Formulation 22](#_Toc415569545)

[5.1.2. Packaging and Labeling 22](#_Toc415569546)

[5.1.3. Storage and Handling 22](#_Toc415569547)

[5.1.4. Dosage and Administration of LDV/SOF 23](#_Toc415569548)

[5.2. Prior and Concomitant Medications 23](#_Toc415569549)

[5.3. Study Drug Adherence 24](#_Toc415569550)

[5.4. Accountability for Study Drug 25](#_Toc415569551)

[5.4.1. Investigational Medicinal Product Return or Disposal 25](#_Toc415569552)

[6. STUDY PROCEDURES 26](#_Toc415569553)

[6.1. Subject Enrollment and Treatment Assignment 26](#_Toc415569554)

[6.2. Pretreatment Assessments 26](#_Toc415569555)

[6.2.1. Screening Visits 26](#_Toc415569556)

[6.2.2. Enrollment Visit 26](#_Toc415569557)

[6.3. Weekly Treatment Visits **Error! Bookmark not defined.**](#_Toc415569558)28

[6.4. Early Termination (ET) 28](#_Toc415569559)

[6.5. Unscheduled Visit 28](#_Toc415569560)

[6.6. Post Treatment Assessments 28](#_Toc415569561)

6.7. Audio Computer Assisted Self Interview (ACASI) 29

[6.8. Assessments for Premature Discontinuation from Study 28](#_Toc415569562)

[6.9. End of Study 29](#_Toc415569563)

[6.10. Procedures and Specifications 29](#_Toc415569564)

[6.10.1. Clinical Laboratory Analytes 29](#_Toc415569565)

[6.10.2. Medical History 29](#_Toc415569566)

[6.10.3. Complete Physical Examination 29](#_Toc415569567)

[6.10.4. Vital Signs 29](#_Toc415569568)

[6.10.5. Creatinine Clearance 30](#_Toc415569569)

[6.10.6. Body Mass Index (BMI) 30](#_Toc415569570)

[6.10.7. Fibrosis-4 Score (FIB-4) 30](#_Toc415569570)

[6.10.8. Fibrosis-Cirrhosis Index (FCI) 30](#_Toc415569570)2

[6.10.9. Viral](#_Toc415569572) RNA Sequencing / Phenotyping Sample 32

[6.10.10 Pregnancy Testing **Error! Bookmark not defined.**](#_Toc415569573)32

6.10.11 Dried blood spots 32

[6.10.12 Quality of Life Surveys 31](#_Toc415569575)3

[6.10.13 Depression Rating Scale 31](#_Toc415569576)3

[6.10.14 Severity of Dependence Scale 31](#_Toc415569577)3

[7. TOXICITY MANAGEMENT 33](#_Toc415569578)

[7.1. Subject Stopping Rules 33](#_Toc415569579)

[8. ADVERSE EVENTS MANAGEMENT 34](#_Toc415569580)

[8.1. Definition of Adverse Events, Adverse Reactions, and Serious Adverse Events 34](#_Toc415569581)

[8.1.1. Adverse Event 34](#_Toc415569582)

[8.1.2. Serious Adverse Events 35](#_Toc415569583)

[8.1.3. Clinical Laboratory Abnormalities and Other Abnormal Assessments as Adverse Events or Serious Adverse Events 35](#_Toc415569584)

[8.2. Assessment of Adverse Events and Serious Adverse Events 36](#_Toc415569585)

[8.2.1. Assessment of Causality for Study Drug and Procedures 36](#_Toc415569586)

[8.3. Assessment of Severity 37](#_Toc415569587)

[8.4. Investigator Requirements and Instructions for Reporting Adverse Events and Serious Adverse Events to Gilead 37](#_Toc415569588)

[8.5. Special Situations Reports **Error! Bookmark not defined.**](#_Toc415569590)39

[8.5.1. Definitions of Special Situations 38](#_Toc415569591)

[8.5.2. Instructions for Reporting Special Situations 38](#_Toc415569592)

[9. STATISTICAL CONSIDERATIONS 40](#_Toc415569593)

[9.1. Analysis Objectives and Endpoints 40](#_Toc415569594)

[9.1.1. Analysis Objectives 40](#_Toc415569595)

[9.1.2. Primary Endpoint 40](#_Toc415569596)

[9.1.3. Secondary Endpoints 41](#_Toc415569597)

[9.1.5. Other Endpoints of Interest 41](#_Toc415569599)

[9.2. Analysis Conventions 41](#_Toc415569600)

[9.4. Demographic Data and Baseline Characteristics 41](#_Toc415569603)

[9.5. Data Analysis 42](#_Toc415569604)

[9.5.1. Primary Analyses 42](#_Toc415569605)

9.5.2 Exploratory Analyses 44

[9.7. Sample Size 44](#_Toc415569612)

[10. RESPONSIBILITIES 45](#_Toc415569613)

[10.1. Investigator Responsibilities 45](#_Toc415569614)

[10.1.1. Good Clinical Practice 45](#_Toc415569615)

[10.1.2. Institutional Review Board (IRB)/Independent Ethics Committee (IEC) Approval 45](#_Toc415569616)

[10.1.3. Informed Consent 45](#_Toc415569617)

[10.1.4. Confidentiality 46](#_Toc415569618)

[10.1.5. Study Files and Retention of Records 46](#_Toc415569619)

[10.1.6. Case Report Forms 47](#_Toc415569620)

[10.1.7. Investigational Medicinal Product Accountability and Return 47](#_Toc415569621)

[10.1.8. Inspections 47](#_Toc415569622)

[10.1.9. Protocol Compliance 47](#_Toc415569623)

[10.2. Sponsor Responsibilities 47](#_Toc415569624)

[10.2.1. Protocol Modifications 47](#_Toc415569625)

[10.2.2. Study Report and Publications 48](#_Toc415569626)

[10.3. Joint Investigator/Sponsor Responsibilities 48](#_Toc415569627)

[10.3.1. Payment Reporting 48](#_Toc415569628)

[10.3.2. Access to Information for Monitoring 48](#_Toc415569629)

[10.3.3. Access to Information for Auditing or Inspections 48](#_Toc415569630)

[10.3.4. Study Discontinuation 48](#_Toc415569631)

[11. REFERENCES 49](#_Toc415569632)

[12. APPENDICES **Error! Bookmark not defined.**](#_Toc415569633)

[Appendix 1. Study Procedures Table **Error! Bookmark not defined.**](#_Toc430780176)

[Appendix 2. Estimating Severity Grade for Parameters Not Identified in the Grading Table 54](#_Toc430780178)5

LIST OF IN-TEXT TABLES

[Table 5‑1. Concomitant Medications Disallowed or to be used with Caution 24](#_Toc430780179)

PROTOCOL SYNOPSIS

San Francisco Department of Public Health

25 Van Ness Ave, Suite 500

San Francisco CA 94102

| Study Title: | Pilot Treatment as Prevention for HCV Among Persons Who Actively Inject Drugs |
| --- | --- |
| IND Number: | N/A |
| Study Centers Planned: | 1 site in the United States |
| Number of Subjects Planned: | 30 subjects |
| Target Population: | Hepatitis C virus (HCV), Genotype 1 infected adults |
| Duration of Treatment: | Subjects will be treated for 8 weeks |
| Objectives: | The primary objectives of this study are as follows:   1. To assess the feasibility of treating persons who actively inject drugs (PWIDs) for HCV with Ledipasvir/Sofosbuvir Fixed-Dose Combination (LDV/SOF) by modified directly observed therapy (mDOT) versus unobserved dosing, with motivational interviewing (MI) adherence counseling, as measured by recruitment and retention rates, overall and by arm. 2. To evaluate the acceptability of mDOT versus unobserved dosing, with MI adherence counseling, among PWIDs treated with LDV/SOF, as determined by mDOT and MI participation rates, medication adherence rates and the proportion of subjects that complete therapy, overall and by arm. 3. To assess through in-depth, semi-structured qualitative interviews, the challenges required for mDOT and unobserved dosing, and identify key factors affecting treatment adherence to LDV/SOF for PWIDs.   The exploratory objectives of this study are as follows:   1. To assess end-of-treatment response and sustained virologic response (SVR), as determined by the proportion with undetectable HCV RNA at week 8 and post-treatment week 12, by arm. 2. To assess adherence to treatment by SOF metabolite levels, by arm, as needed based on SVR rates and medication adherence data. 3. To assess drug use behaviors before and after HCV treatment. 4. To assess HCV relapse and reinfection at post-treatment week 36 among participants achieving SVR. 5. To evaluate demographic, behavioral and HCV strain correlates of adherence and SVR. 6. To characterize the social and injector networks of participants in preparation for a subsequent full TasP trial. |
| Study Design: | Thirty subjects will be enrolled 2-to-1 in one of the following two treatment arms:  **Group 1:** Modified Directly Observed Therapy (N=20)   - LDV/SOF tablet (LDV 90mg/SOF 400mg) observed daily dosing (modified for non-observed Saturday and Sunday dosing) for 8 weeks   **Group 2:** Unobserved Dosing (N=10)   - LDV/SOF tablet (LDV 90mg/SOF 400mg) provided weekly (7 tablets) for unobserved daily dosing for 8 weeks |
| **Diagnosis and Main Eligibility Criteria:** | Genotype 1, HCV infected, male and non‑pregnant/non‑lactating female subjects, ages 18 years or older, treatment-naïve, and injection drug use in past 30 days.  Reference Section 4.2 and 4.3 of the protocol for detailed Inclusion and Exclusion criteria. |
| - 1. **Study Procedures/ Frequency:** | Screening assessments will be completed across two visits within 10 days of each other and not more than 30 days prior to the enrollment visit.  All subjects will complete the following study visits: screening 1 and 2, enrollment, study visit at weeks 1, 2, 3, 5, 6 and 7, in-depth study visit at weeks 4 and 8, and post-treatment visits at 1, 12 and 36 weeks following the last dose of study drug. For mDOT subjects, there will be additional on-site visits Monday–Friday from enrollment through the week 8 visit for observed dosing.  After consent is obtained and eligibility is determined, screening assessments (split between two visits) will include medical history and physical examination (including weight and height), review of systems, vital signs, concomitant medications, CBC with diff/CMP, PT/INR, HBsAg, HCV RNA, HCV genotype and sub-type (or lab documentation during period of diagnosis of chronic HCV), urine drug screen, HIV Ag/Ab test, and stored sample for potential future.  Enrollment visit will include a urine drug screen, urine β‑hCG (females of childbearing potential only), vital signs, randomization to study arm, motivational interviewing–based counseling pertaining to risk reduction and medication adherence, administration of the Audio Computer Assisted Self Interview (ACASI), and medication dispensation (unobserved dosing) or first directly observed dosing (mDOT).  Study visits (weeks 1, 2, 3, 5, 6, 7) will include directly observed dosing (mDOT) or medication dispensation (unobserved dosing), study drug dosing compliance (pill count), review of concomitant medications and adverse events, urine drug screen, stored samples for potential testing of SOF and LDV metabolite level, and early termination procedures (if applicable). CBC with diff/CMP and HCV RNA will take place at week 2.  Week 4 visit will include all procedures for weekly study visits as well as symptom-driven physical examination, vital signs, ACASI, HCV RNA, and motivational interviewing for medication adherence.  Week 8 visit will include all procedures for weekly study visits as well as symptom-driven physical examination, vital signs, HCV RNA, CBC with diff/CMP, HIV Ag/Ab, ACASI, and a qualitative interview. |
|  | Week 1 post-treatment visit will include review of adverse events, provision of end-of-treatment HCV RNA results, and motivational interviewing–based risk reduction counseling. The rationale for the post-treatment week 1 visit is to inform subjects of their SVR status in person (as opposed to via phone) and provide risk-reduction counseling immediately thereafter. Week 8 is too lengthy to provide SVR status and comprehensive risk-reduction counseling in addition to the other week 8 procedures.  Weeks 12 and 36 post-treatment visits will include review of adverse events, urine drug screen, ACASI, HCV RNA, stored samples for potential HCV RNA sequencing, motivational interviewing-based risk reduction counseling (week 12).  The ACASI, which is administered at enrollment, weeks 4, 8, and post-treatment weeks 12 and 36, will include injection drug use and injection risk behavior, partner-by-partner data on injection/substance use/HCV status, demographic information (enrollment only), drug use history (enrollment only), quality of life scale (EQ5-D), Center for Epidemiologic Studies Depression Rating Scale, and Severity of Dependence Scale and participant satisfaction with treatment (week 8 only). |
| Test Product, Dose, and Mode of Administration: | LDV/SOF is manufactured as a fixed dose combination tablet, consisting of 90 mg LDV and 400 mg SOF, for oral administration. Subjects will take 1 tablet daily with or without food. |
| Reference Therapy, Dose, and Mode of Administration: | mDOT subjects will take 1 tablet daily on-site directly observed by study clinician or specifically trained and authorized study staff. Each Friday, mDOT subjects will receive 2 doses in a WisePill device to be taken Saturday and Sunday unobserved.  Unobserved dosing subjects will receive 7 tablets weekly in a WisePill device to be taken daily unobserved.  Both arms will take 1 tablet orally daily. |
| Criteria for Evaluation |  |
| **Safety:** | AEs and safety laboratory tests will be collected throughout the study (including post-treatment weeks 1, 12 and 36). |
| **Efficacy:** | Efficacy will be evaluated using scheduled assessments of HCV RNA performed using the Roche TaqMan HCV Quantitative Assay. |
| **Pharmacokinetic:** | A single pharmacokinetic (PK) blood sample will be collected and weeks 2, 4, and 8, and early termination (if applicable) for all subjects. PK studies will be run as needed based on end-of-treatment and SVR rates.  The PK of sofosbuvir (and its metabolites GS‑566500 and GS‑331007) and ledipasvir may be assessed. |
| Statistical Methods: | **Aim 1**: To assess the feasibility of treating active PWIDs for HCV with LDV/SOF by mDOT versus unobserved dosing, as measured by recruitment and retention rates overall and by arm. We will calculate the following feasibility measures: 1) proportion of those eligible among those screened; 2) proportion enrolled among those eligible; and 3) study retention and completion rates, overall and by arm. Process measures (e.g., visit length) and reactions to study procedures will also be assessed. Descriptive statistics will be presented with 95% confidence intervals, to help in interpretation. Between-group differences in retention and completion will be assessed using Fisher’s exact and Wilcoxon tests. We will also calculate Kaplan-Meier curves for time to dropout, by group, and test for differences using the log-rank test.  **Aim 2**: To evaluate the acceptability of mDOT versus unobserved dosing, the percent of treatment medication adherence to LDV/SOF, as measured by the percent of doses taken overall (observed and unobserved), will be assessed using DOT doses and weekend Wise Pill data for the mDOT arm, and WisePill data for the unobserved dosing arm. Other outcomes of interest will include completion of therapy, patterns of adherence, and time to stopping medication. Concordance of the various adherence measures will be examined using weighted Kappa and intraclass correlations. Between-arm differences will be assessed using Fisher’s exact and Wilcoxon tests for outcomes measured once, and GEE models for repeated outcomes. We will also use GEE models to compare WisePill openings and pill counts, accounting for between-arm differences in expected frequency.  Sample size justification: The sample will provide typical precision for a pilot study of feasibility and acceptability outcomes. Specifically, we will be able to estimate the mean of continuous outcomes within margins of sampling error (MSEs; half-widths of 95% confidence intervals) of 0.37 standard deviations (SD) overall, and 0.55 SDs within arm; percentages will be estimated within MSEs of 11-18 points overall, and 15-25 points within arm, depending on the sample value. In designing the full-scale trial, these exploratory results will be cautiously interpreted in the light of confidence intervals, plausibility, and prior results.  **Aim 3**: To assess through in-depth, semi-structured qualitative interviews, the challenges with time intensity required for mDOT versus unobserved dosing, and identify key factors affecting treatment adherence for PWIDs treated with LDV/SOF. Transcribed semi-structured interviews conducted at the End-of-Treatment (week 8 or early termination) visit will be imported into Atlas.ti, a qualitative research software program. We will utilize two qualitative analytic methods to interpret data. First, content analysis will allow us to focus on answering study questions regarding experiences with LDV/SOF treatment and DOT strategies, and opinions on challenges to achieving consistent adherence to treatment. *A priori* themes of interest primarily focused on the time-intensity required for this treatment plan and based upon the team’s experience with HCV treatment among PWIDs will be developed and used as the first codebook with which data are coded. Second, thematic analysis with open coding will allow themes to emerge from the data. A coding system will be developed using an iterative process. In the initial phase, we will review all transcripts and field notes to become familiar with the data, and the analytic team will make notes of their observations and potential codes for use in the initial analysis meeting. Then a codebook, consisting of the code definition and inclusion and exclusion criteria, will be created to aid analysis. In addition to predetermined HCV treatment dimensions, additional codes will be developed independently by each analytic team member through an inductive process of identifying themes that emerge from the data. Group analysis meetings will be held to compare independently developed codes for similarity and further definition. A consensus will be reached regarding each code, its application and definition. To ensure consistency, a codebook and dictionary will be developed with universal definitions for each code.  *Inter-rater reliability*: To ensure reliability and validity in the open-ended component of the in-depth interviews analysis, we will implement a verification strategy to ensure self-correction throughout the analysis process. The two coders will assess inter-rater reliability by calculating the correlation between a set of ratings done by two independent raters for the initial set of 3-6 transcripts, and then again on the next set of 6 transcripts. After each inter-rater reliability assessment, the codebook and codes will be revised based on reconciliation of findings done between coders. Inter-rater reliability will be assessed as a team twice during the analysis process and again at the end of analysis as a post-hoc evaluation of reliability and validity. This evolving analysis process will ensure high quality of research decisions, the rationale behind decisions, and the responsiveness of investigators to the data. Once the codebook is developed and verified all remaining transcripts will be coded. Further meetings will be held to discuss any differences in coding and to ensure consistency in the application of codes.  Final analysis will begin with data reduction, then display, analysis and conclusion drawing using a conceptual framework in which hypothesized factors affecting adherence are assessed and unanticipated findings are identified. The analytic team will work from the coded data to merge findings into a final summary and a consensus of major themes, relationships between themes, and ranking of items of most importance to adherence. This method of data reduction encompassing both site and team based analysis creates a robust iterative process through which the data is thoroughly discussed and analytical consensus achieved. *Atlas.ti* software will be used to both code and develop data displays used in the analysis for this study.  **Exploratory Aims**: While we recognize the small sample size limits our ability to draw firm conclusions from exploratory analyses, the paucity of data for this treatment approach allows for hypothesis-generating results.  *End-of-treatment response and SVR*: We will compare the proportion of participants with undetectable HCV RNA at week 8 and post-treatment week 12 between arms using Fisher’s exact tests.  *Acceptability based on drug levels*: If SVR, retention and/or medication adherence data suggestion medication compliance issues, we may compare SOF metabolite–positivity rates will be calculated by week in both arms; between-group differences will be compared using GEE models for repeated outcomes.  *HCV relapse and reinfection*: Among participants who achieve SVR, we will determine the proportion who experience HCV relapse and reinfection at post-treatment week 36, overall and by arm, with exact 95% confidence intervals. We will sequence HCV RNA in such cases to establish if the event represents relapse versus reinfection.  *Social and injection network analyses to inform a subsequent full TasP trial*: We will characterize injector network sizes at baseline and follow-up. Newman’s method will be used to calculate assortativity coefficients, a measure of the degree of demographic and risk behavior similarity within networks. These findings will help us determine whether a full efficacy trial should use targeted sampling strategies to ensure diversity in network sizes, and whether randomization should be stratified by one or both of these factors in future trials.  **Other Exploratory Outcomes**:  *Drug use behaviors before and after HCV treatment*: Recent data suggest significant changes in drug use behaviors after HCV diagnosis: serosorting with other HCV-positive PWIDs and a sustained reduction in drug use. We will assess these behaviors at baseline and follow-up for preliminary estimates of the possible impact of HCV treatment on these behaviors. ANCOVA and conditional logistic models will be used for continuous and binary outcomes respectively.  *Correlates of Adherence and SVR*: We will explore demographic, behavioral correlates of medication adherence using GEE models for repeated measures. In addition, we will explore demographic and behavioral correlates of SVR using Fisher exact tests and exact logistic regression. |

This study will be conducted in accordance with the guidelines of Good Clinical Practices (GCPs) including archiving of essential documents.

GLOSSARY OF ABBREVIATIONS AND DEFINITION OF TERMS

| ° C | degrees Celsius |
| --- | --- |
| ° F | degrees Fahrenheit |
| β-hCG | β-human chorionic gonadotropin |
| ACASI | Audio-Computer Assisted Self Interview Software |
| AE | adverse event |
| ANC | absolute neutrophil count |
| ANCOVA | Analysis of covariance |
| ALT | Alanine transaminase |
| AST | aspartate aminotransferase (also SGOT) |
| BMI | body mass index |
| DSMB | Data safety monitoring board |
| DSMP | Data safety monitoring plan |
| BW | body weight |
| CBC | Complete blood count |
| CDC | Center for Disease Control and Prevention |
| CHR | Committee on human research |
| CI | confidence interval |
| CL_cr_ | creatinine clearance |
| CRF | case report form(s) |
| CMP | Comprehensive metabolic panel |
| CRO | Contract (or clinical) research organization |
| DAA | Direct acting antiviral |
| DAIDS | Division of AIDS Table for Grading the Severity of Adult and Pediatric Adverse Events |
| DOT | Directly observed therapy |
| DSPH | Drug Safety and Public Health |
| EOT | End of Treatment |
| ET | Early Termination |
| FDA | (United States) Food and Drug Administration |
| FDC | Fixed-dose combination |
| GCP | Good Clinical Practice (Guidelines) |
| GEE | Generalized estimating equation |
| GSI | Gilead Sciences, Inc. |
| GT | Genotype (viral) |
| Hb | Hemoglobin |

| HBV | Hepatitis B virus |
| --- | --- |
| HCV | Hepatitis C virus |
| HDPE | High-density polyethylene |
| IB | Investigator Brochure |
| IEC | independent ethics committee |
| IMP | Investigational Medical Product |
| INR | International Normalized Ratio of prothrombin time |
| IRB | institutional review board |
| LDV | Ledipasvir |
| LLN | lower limit of the normal range |
| MCV | mean corpuscular volume or mean cell volume |
| mDOT | Modified directly observed therapy |
| MI | Motivational interviewing |
| min | Minute |
| mmHg | millimeters mercury |
| MSE | Mean squared error |
| PI | Principal investigator |
| PO | per os, by mouth or orally |

| PT | Prothrombin time |
| --- | --- |
| PWID | People who inject drugs |
| QD | once daily (use only in tables) |
| RBC | Red blood cells |
| RBC | red blood cell count |
| SADR | serious adverse drug reaction |
| SAETRS | Serious Events Tracking and Reporting Systems |
| SAMHSA | Substance Abuse and Mental Health Services |
| Scr | serum creatinine |
| SAE | serious adverse event |
| SD | standard deviation |
| SOF | Sofosbuvir |
| SOP | standard operating procedure |
| SUSAR | suspected unexpected serious adverse reaction |
| SVR | sustained virologic response |
| TasP | Treatment as Prevention |
| UCSF | University of California, San Francisco |
| UCLA | University of California, Los Angeles |
| ULN | upper limit of the normal range |
| US | United States |
| VL | Viral load |
| WBC | white blood cells |

# INTRODUCTION

## Background

Worldwide, 170 million people are infected with Hepatitis C (HCV).[^1^](#_ENREF_1) Persons who inject drugs (PWIDs) have the highest burden of HCV in the US and globally. In the US, an estimated 43% of PWIDs have chronic HCV and National HIV Behavioral Surveillance data suggest that 50-60% of all PWIDs have HCV, notwithstanding high levels of sterile syringe use.[^2^](#_ENREF_2) The Centers for Disease Control and Prevention (CDC) estimates at least 18,000 new HCV infections per year, almost exclusively among PWIDs, with a 44% increase in the number of acute cases from 2010 to 2011 and a 50% increase through 2012. The CDC now considers there to be an emerging epidemic of HCV among young PWIDs, due in part to expansions and retractions in opioid prescribing and related transitions to injection drug use that have occurred in communities across the US.[^3^](#_ENREF_3) In San Francisco, HCV incidence among young adult PWIDs is 26%.[^4^](#_ENREF_4) While the hepatic sequelae of HCV may take decades to develop, ongoing use of injection drugs is the principal source of secondary transmission.

Mathematical models suggest that treating PWIDs for HCV could result in substantial reductions in both prevalence and incidence of HCV. “Treatment as Prevention” (TasP), is an approach to prevention whereby treatment is used to decrease the chance of forward transmission. Treating 8% of PWIDs annually for HCV could reduce HCV prevalence—the major driver for incidence—by 90% over 15 years according to a conservative TasP model that assumed that re-infection rates post-treatment were the same as initial infection rates.[^5^](#_ENREF_5) Cohort studies, however, estimate the re-infection rate at 4-6% per year[^6^](#_ENREF_6)^,^[^7^](#_ENREF_7), about half the rate assumed for initial infection, and a study of PWIDs treated with interferon-based therapy demonstrated a re-infection rate of 2.8%[^8^](#_ENREF_8); thus, the impact of TasP may be greater than predicted. An analysis of the use of novel HCV therapeutics found that treating HCV-infected persons will *fail to impact incidence unless delivered to those with early-stage infection*; this is because very few of those at risk of secondary transmission have advanced liver disease.[^9^](#_ENREF_9) Thus, treating to prevent HCV incidence is distinct from treating to prevent hepatic sequelae.

## Ledipasvir/Sofosbuvir Fixed-Dose Combination

LDV/SOF (also known as SOF/LDV) fixed-dose combination combines two HCV specific direct acting antiviral (DAA) agents into a single tablet for the treatment of chronic HCV infection.

## Rationale for the Current Study

Directly observed therapy (DOT) strategies have only recently been evaluated for HCV treatment—to date, data among substance users are promising but involve only interferon-based therapies. A pilot study of pegylated interferon injections administered in a methadone clinic with self-administered twice-daily ribavirin found 100% compliance and high SVR[^10^](#_ENREF_10), as did daily DOT in methadone and buprenorphine treatment programs[^11^](#_ENREF_11). A randomized trial of DOT (for interferon injections only) versus self-administration of all medications among methadone patients found higher SVR rates among those receiving DOT (54% vs 33%). A pilot study of DOT for weekly interferon injections among out-of-treatment PWIDs demonstrated SVR of 58% and no associations between past month drug use and completion of HCV therapy or SVR[^8^](#_ENREF_8). These results suggest that DOT may be beneficial, at least for interferon-based regimens, and that ongoing drug use may not be a significant deterrent to successful HCV treatment. These strategies remain underexplored among actively using PWIDs, the most efficient target group for a TasP initiative, and have not yet been tested for novel HCV therapies.

This study will test the acceptability of a once daily DAA regimen for HCV among actively injecting PWIDs. Active substance users have been excluded from clinical trials of DAAs. Given the high cost of LDV/SOF ($1200/pill) and the limits already placed by insurers (such as 6-12 months of abstinence prior to treatment and advanced fibrosis), access to DAAs for PWIDs is likely to remain extremely limited for the near future. This study will assess the acceptability of HCV therapy with DAAs among PWIDs, necessary data not only for future TasP interventions, but also for efforts to improve HCV clinical care for PWIDs.

## Rationale for Dose Selection of Ledipasvir/Sofosbuvir Fixed Dose Combination

LDV/SOF tablet (90 mg/400 mg) administered with or without RBV has demonstrated favorable safety and efficacy profiles in over 3000 HCV‑infected subjects across different patient populations in Phase 2 and 3 trials. These doses represent the marketed doses of ledipasvir and sofosbuvir.

## Overall Risk/Benefit Assessment

This study aims to provide an effective treatment option to PWIDs with HCV infection who currently lack effective treatment options due to their ongoing injection drug use. The results of this study will be used to develop the optimal route of medication delivery for future interventions aiming to treat HCV among persons actively injecting drugs.

The study will be evaluated on an ongoing basis by the UCLA Addiction Medicine Data Safety Monitoring Board.

## Compliance

This study will be conducted in compliance with this protocol, Good Clinical Practice (GCP), and all applicable regulatory requirements.

# OBJECTIVES

The primary objectives of this study are:

- To assess the feasibility of treating active PWIDs for HCV with LDV/SOF by mDOT versus unobserved dosing, with MI adherence counseling, as measured by recruitment and retention rates, overall and by arm.
- To evaluate the acceptability of mDOT versus unobserved dosing, with MI adherence support, among PWIDs treated with LDV/SOF, as determined by mDOT and MI participation rates, medication adherence rates and the proportion of subjects that complete therapy, by arm.
- To assess through in-depth, semi-structured qualitative interviews, the challenges with time intensity required for mDOT and unobserved dosing interventions, and identify key factors affecting treatment adherence for PWIDs treated with LDV/SOF.

Exploratory objectives of this study are:

- To assess end-of-treatment response and SVR, as determined by the proportion with undetectable HCV RNA at weeks 8 and post-treatment week 12, by arm.
- To potentially assess adherence to treatment by weekly SOF metabolite levels, by arm (depending on SVR results) as applicable.
- To assess drug use behaviors before and after HCV treatment.
- To assess HCV relapse and reinfection at post-treatment week 36 among participants achieving SVR.
- To evaluate demographic, behavioral and HCV strain correlates of adherence and SVR.
- To characterize the social and PWID networks of participants in preparation for a subsequent full TasP trial.

# STUDY DESIGN

## Study Treatment and Duration of Treatment

This is an open-labeled study that will evaluate the feasibility, acceptability, and effectiveness of mDOT versus unobserved dosing of 8 weeks of LDV/SOF in genotype 1 HCV–infected persons who actively inject drugs.

Thirty subjects will be enrolled in one of the following two treatment groups.

**Group 1**: Subjects receiving modified directly observed therapy

- - - LDV/SOF tablet (LDV 90mg/SOF 400mg) once daily for 8 weeks (dosing observed daily Monday-Friday).

**Group 2**: Subjects receiving unobserved weekly dosing

- - - LDV/SOF tablet (LDV 90mg/SOF 400mg) once daily for 8 weeks (tablets dispensed weekly and dosing is unobserved).

## Treatment Discontinuation Criteria

Study drug will be discontinued in the following instances:

- Unacceptable toxicity, as defined in Section 7 of the protocol, or toxicity that, in the judgment of the investigator, compromises the ability to continue study-specific procedures or is considered to not be in the subject’s best interest
- Subject request to discontinue for any reason; it is important to determine whether the withdrawal of consent is primarily due to an AE, lack of efficacy, or other reason
- Discontinuation of the study at the request of Gilead, regulatory agency, or an Institutional Review Board (IRB), with appropriate justification

Study drug *may* be discontinued based on individual case review by both study physicians in the event of pregnancy of a female subject.

If a subject meets discontinuation criteria during treatment, an early termination (ET) visit will be required (Section 6.4). All subjects who meet discontinuation criteria will complete an ET visit, which will include all week 8 procedures, and post-treatment visits at weeks 1, 12 and 36, regardless of the treatment duration.

# SUBJECT POPULATION

## Number of Subjects and Subject Selection

Thirty (30) subjects will be enrolled in this study.

At this time, thirty subjects have been successfully enrolled. There is enough study drug left to enroll one more subject in this study. Since there is enough study drug to treat someone, an extra study subject will be enrolled and will follow the same protocol guidelines as the previous subjects.

## Inclusion Criteria

Subjects must meet *all* of the following inclusion criteria to be eligible for participation in this study.

**(1) ≥**18 years of age;

**(2)** 2 consecutive positive HCV RNA tests at least 6 months apart;

**(3)** HCV genotype 1;

**(4)** HCV RNA <6 million copies by Roche TaqMan® Assay

**(5)** No evidence of hepatic cirrhosis (as determined by two indices: Fib4 <3.25 and fibrosis-cirrhosis index (FCI) <1.25;

**(6)** Drug injection in past 30 days by self-report *and* physical exam evidence of injection drug use (e.g., track marks);

**(7)** Injected with others in past 12 months by self-report;

**(8)** Lab values within acceptable range (platelets >50,000, creatinine clearance by Cockroft-Gault ≥60mL/min, hemoglobin >10g/dL, INR <1.5 x upper limit of normal (ULN) unless stable on anticoagulant regimen or known hemophilia, AST/ALT <10 x ULN);

**(9)** Able to speak English;

**(10)** No plans to leave the San Francisco area for at least 9 months and either lives or works in San Francisco, or travels to San Francisco at least weekly;

**(11)** For women of childbearing age, pregnancy test negative, not actively nursing, and agree to use birth control during treatment (although LDV/SOF has a FDA Pregnancy Category rating of B, consistent with no known evidence of harm, treatment is not urgent for these patients so we will err on the side of caution).

## Exclusion Criteria

Subjects who meet *any* of the following exclusion criteria are not to be enrolled in this study.

**(1)** HIV+;

**(2)** HBV surface antigen +;

**(3)** Non-definitive HCV genotype results;

**(4)** Previously received treatment for HCV (interferon, ribavirin, or DAA);

**(5)** Taking medications that affect pharmacokinetics of LDV/SOF (proton-pump inhibitors, anticonvulsants [phenobarbital, phenytoin, carbamazepine, oxycarbazepine], rifamycins, rosuvastatin, herbs [St. John’s wort, silymarin, echinacea]);

**(6)** History of any of the following:

- 1. Current gastrointestinal disorder or post-operative condition that could interfere with the absorption of the study drug
  2. History of hepatic decompensation (i.e., ascites, encephalopathy or variceal hemorrhage)
  3. History of solid organ or bone marrow transplantation
  4. Current treatment for cancer

**(7)** Chronic liver disease for non‑HCV reason (e.g., Wilson’s disease, alpha‑1 antitrypsin deficiency, cholangitis) except iron overload;

**(8)** Use of any prohibited concomitant medications as described in Section 5.2 within 21 days of the enrollment visit; and

**(9)** Known hypersensitivity to LDV, SOF, the metabolites, or formulation excipients.

**(10)** Any other conditions that preclude study involvement as determined by PI.

# INVESTIGATIONAL MEDICINAL PRODUCTS

## Description and Handling of LDV/SOF

### Formulation

LDV/SOF tablets are orange, diamond-shaped, film-coated tablets containing 90 mg of LDV and 400 mg of SOF. The tablets are debossed with “GSI” on one side and “7985” on the other side. The LDV/SOF tablets contain the following inactive ingredients: lactose monohydrate, copovidone, microcrystalline cellulose, croscarmellose sodium, colloidal silicon dioxide, magnesium stearate, polyvinyl alcohol, titanium dioxide, talc, polyethylene glycol, FD&C yellow # 6/sunset yellow FCF aluminium lake.

### Packaging and Labeling

LDV/SOF tablets are packaged in white, high-density polyethylene (HDPE) bottles. Each bottle contains 30 tablets, a silica gel desiccant canister or sachet and polyester packing material. Each bottle is enclosed with a white, continuous thread, child‑resistant screw cap with an induction-sealed, aluminum‑faced liner.

All labels for LDV/SOF bottles to be distributed to centers in the US shall be labeled to meet all applicable requirements of the US Food and Drug Administration (FDA) and/or other local regulations as applicable.

Sufficient quantities of LDV/SOF tablets to complete the entire study will be shipped to the investigator or qualified designee from Gilead Sciences Materials & Logistics (or its designee).

### Storage and Handling

LDV/SOF bottles will be stored at controlled room temperature until required for administration. Controlled room temperature is defined as 25 °C (77 °F); excursions are permitted between 15 °C and 30 °C (59 °F to 86 °F).

All drug products will be stored in a securely locked area, accessible only to authorized site personnel. To ensure the stability of the study drug and to ensure proper product identification, the drug product will not be stored in a container other than the container in which they are supplied. Consideration will be given to handling, preparation, and disposal through measures that minimize drug contact with the body. Appropriate precautions will be followed to avoid direct eye contact or exposure through inhalation when handling LDV/SOF.

LDV/SOF pills will be repackaged according to defined standard operating procedures. Study pills will be transferred to WisePill containers in our on-site pharmacy only once a subject has arrived for a study visit. The pills will be transferred by study clinicians using gloves and designated pill tongs. Pills will be double counted to assure that there are no errors in the pill dispensation.

### Dosage and Administration of LDV/SOF

LDV/SOF tablet is to be administered once daily with or without food. Each subject will be instructed to maintain approximately the same daily dosing interval between study drug doses.

For missed dose(s) of study drug, subjects will be instructed to take the missed dose(s) of study drug as soon as possible during the same day. Subjects will be cautioned never to double the next dose with a missed dose of study drug under any circumstances.

mDOT study participants will choose a daily 3 hour “window” between 8:00AM–4:00PM in which they are expected to arrive at the study site to receive their daily dose of LDV/SOF. Subjects who have not arrived at the study site 2 hours into their designated “window” will receive one reminder (via phone call, text or email, based on subject preference). If a subject misses a daily visit, s/he will be advised to come to the study site as early as possible the following day. If the subject arrives within 18 hours of his/her designated dispensing time, a dose of LDV/SOF will be dispensed and the subject will be told s/he can return the same day for an additional dose at a minimum of 8 hours after the off-schedule dose was dispensed. In this case, the subject would resume his/her standard schedule the subsequent day.

Study staff will help subjects arrange a personal automated reminder message via their own phone, email account, or other device/service as a daily reminder to take medication (unobserved arm) or come to the study site (mDOT).

In the event that a subject in either arm anticipates missing an upcoming visit, protocol will allow for the subject to receive tablets up to 7 days beyond their planned dispensing dose.

In the event of lost or stolen medication, subjects can receive up to a maximum of 7 replacement tablets.

In the event that a subject in either arm is unable to attend study visits (due to hospitalization, detainment, or incarceration), protocol will allow for study staff to deliver medication to subject whenever possible. In such a case, subjects would receive up to 7 tablets of LDV/SOF per drop-off for unobserved daily dosing regardless of study arm.

Medication for unobserved dosing will always be dispensed in a Wisepill container unless not permitted by the facility in which subject resides.

## Prior and Concomitant Medications

Concomitant medications taken within 30 days prior to screening will be recorded in the source documents and case report form(s) (CRFs).

Concomitant use of certain medications or herbal/natural supplements (inhibitors or inducers of drug transporters i.e., P‑gp) with study drug may result in PK interactions increasing or decreasing exposure to study drug.  Examples of representative medications which are prohibited from 21 days prior to enrollment through the end of treatment are listed below. The use of amiodarone is prohibited from **60 days prior to enrollment** through the end of treatment. Participants will be advised to avoid selected medications until 21 days after the end of treatment (e.g. digoxin and rosuvastatin)

Table 5‑1. Concomitant Medications Disallowed or to be used with Caution

| Drug Class | Agents Disallowed | Use with Caution |
| --- | --- | --- |
| Acid Reducing Agents^a^ |  | Proton- Pump Inhibitors, H2‑Receptor Antagonists, Antacids |
| Anticonvulsants^b^ | Phenobarbital, Phenytoin, Carbamazepine, Oxcarbazepine |  |
| Antimycobacterials^b^ | Rifabutin, Rifapentine, Rifampin |  |
| Cardiac Medications | Amiodarone^c^ | Digoxin^d^ |
| Herbal/Natural Supplements^b^ | St. John’s Wort, Echinaccea, Milk thistle (i.e., silymarin), Chinese herb sho-saiko-to (or Xiao-Shai-Hu-Tang) |  |
| HMG-CoA Reductase Inhibitors^e^ | Rosuvastatin |  |

a It is recommended to separate antacid and LDV/SOF administration by 4 hours. H2-receptor antagonists may be administered simultaneously with or staggered from LDV/SOF at a dose that does not exceed doses comparable to famotidine 40 mg twice daily. Proton-pump inhibitor doses comparable to omeprazole 20 mg can be administered simultaneously with LDV/SOF. Proton-pump inhibitors should not be taken before LDV/SOF.

b May result in a decrease in the concentrations of study drug.

c May result in symptomatic bradycardia. Mechanism is not currently known. The use of amiodarone is prohibited from 60 days prior to Baseline/Day 1 through the end of treatment.

d May result in an increase in the concentration of study drug and/or concomitant medications. Co-administration of LDV/SOF with digoxin may increase the concentration of digoxin. Caution is warranted and therapeutic concentration monitoring of digoxin is recommended when co-administered with LDV/SOF.

e Use with study drug may result in an increase in the concentration of rosuvastatin. Monitor for signs and symptoms of muscle weakness or myopathy, including rhabdomyolysis.

Medications for disease conditions **excluded** from the protocol (e.g., active cancer, organ transplantation) are not listed under this Concomitant Medication section and are disallowed in the study.

## Study Drug Adherence

mDOT: Subjects will be observed taking LDV/SOF on a daily basis Monday through Friday. Two take-home tablets (doses for Saturday and Sunday) will be dispensed on Friday and will be monitored by the use of a Wise Pill Dispenser, a device currently used in our other trials, which allows for real-time transmission of opening data to estimate medication adherence. Wise Pill Dispensers have been shown to be reliable for real time monitoring of medication adherence, even in resource-limited settings.

Unobserved dosing: Subjects will be provided 7 tablets of LDV/SOF once weekly to take home in a WisePill Dispenser. Medication compliance of study drug will be assessed at every post-enrollment visit through weekly pill counts conducted by study staff. The pill count will take place in the on-site pharmacy and will occur prior to that week’s pill dispensation. If medication compliance is low, study staff will offer basic medication adherence advice to the subject.

## Accountability for Study Drug

The investigator is responsible for ensuring adequate accountability of all used and unused study drug bottles. This includes acknowledgement of receipt of each shipment of study drug (quantity and condition).

LDV/SOF accountability records will:

- Record the date received and quantity of study drug shipment
- Record the date, subject number, subject initials, the study drug bottle number dispensed
- Record the date, quantity of used and unused study drug returned, along with the initials of the person recording the information.

Subjects will be instructed to bring back all study drug at every post‑enrollment study visit through the end of treatment.

### Investigational Medicinal Product Return or Disposal

Please refer to Section 10.1.7 for Investigational Medicinal Product Accountability and Return.

# STUDY PROCEDURES

The study procedures to be conducted for each subject enrolled in the study are presented in tabular form in Appendix  and described in the text that follows. Additional information is provided in the study procedures manual.

The investigator will document any deviation from protocol procedures and notify appropriate regulatory authorities (e.g., IRB, DSMB, NIH, and/or Gilead).

## Subject Enrollment and Treatment Assignment

Screening and on-treatment visits will occur at screening visits 1 and 2, enrollment, and weeks 1-8. Post-treatment visits will be scheduled at weeks 1, 12 and 36 after the date of last administration of study drug.

All enrolled subjects will complete week 8 or early termination, and post-treatment weeks 1, 12 and 36 visits regardless of the treatment duration.

## Pretreatment Assessments

### Screening Visits

Subjects will be screened within 30 days of enrollment to determine eligibility for participation in the study. The screening window can be extended to 42 days in exceptional circumstances. Subjects meeting all of the inclusion criteria and none of the exclusion criteria will return to the clinic within 30 days after screening for enrollment into the study.

After consent is obtained and eligibility is determined, screening assessments (split between two visits) will include medical history and physical examination (including weight and height), review of systems, vital signs, concomitant medications, CBC with diff/CMP, PT/INR, HBsAg, HCV RNA, HCV genotype and sub-type (or lab documentation during period of diagnosis of chronic HCV), urine drug screen, HIV Ag/Ab test, and stored sample for potential HCV sequence analysis.

Subjects will be compensated $15 for screen 1 and $15 for screen 2.

### Enrollment Visit

Enrollment tests and procedures will be completed prior to enrollment and dosing/dispensing of study drug.

Enrollment visit will include a urine drug screen, urine β‑hCG (females of childbearing potential only), vital signs, randomization to study arm, motivational interviewing–based counseling pertaining to risk reduction and medication adherence, administration of the Audio Computer Assisted Self Interview (ACASI), and medication dispensation (unobserved dosing) or first directly observed dosing (mDOT).

Subjects will be randomized 2:1 to mDOT (n=20) or unobserved dosing (n=10) using permuted blocks of randomly selected sizes 2 and 4 generated using a STATA computer program. Staff members who have no contact with study participants will enclose and seal treatment allocation cards in sequentially numbered opaque envelopes to correspond with the randomization sequence. As a participant is enrolled, the envelope that corresponds with their sequence will be opened to reveal their randomized treatment condition.

mDOT subjects will receive a 10-trip Muni card to cover weekly round-trip transportation to study site and the unobserved dosing arm will receive a weekly two-trip Muni card to cover round-trip transportation to study site. These cards will be dispensed at weekly visits only so as to avoid directly compensating participants for attending daily visits.

Subjects will be compensated $20 for the enrollment visit.

## Weekly Treatment Visits (weeks 1-8)

On-treatment study visits (weeks 1, 2, 3, 5, 6, 7) will include directly observed dosing (mDOT) or medication dispensation (unobserved dosing), study drug dosing compliance (pill count), review of concomitant medications and adverse events, urine drug screen, and stored samples for potential testing of SOF and LDV metabolite levels and HCV sequencing, and early termination procedures (if applicable). CBC with diff/CMP and HCV RNA will take place at week 2.

Week 4 visit will include all procedures for weekly study visits as well as symptom-driven physical examination, vital signs, ACASI, HCV RNA, and motivational interviewing for medication adherence.

Week 8 visit will include all procedures for weekly study visits as well as symptom-driven physical examination, vital signs, HCV RNA, CBC with diff/CMP, HCV RNA, HIV Ag/Ab, ACASI, and a qualitative interview.

At each weekly visit, mDOT subjects will receive a 10-trip Muni card to cover weekly round-trip transportation to study site and the unobserved dosing arm will receive a weekly two-trip Muni card to cover round-trip transportation to study site.

Subjects will be compensated $20 for weekly visits 1, 3, 5, 6, and 7; and $25 for the weekly visits with blood draws and/or longer procedures, weeks 2, 4 and 8.

## Early Termination (ET)

For subjects who terminate early, the ET visit will include the same procedures for a standard week 8 visit. Participants will then be asked to complete post-treatment weeks 1, 12 and 36 starting after last dose of the study drug (post-treatment week 1 visit will be 1 week after ET visit).

Gilead (e.g., Medical Monitor and Clinical Program Manager)/CRO will be informed as soon as possible when a subject discontinues treatment.

## Unscheduled Visit

A subject should attend an unscheduled visit if requested by the investigator. The assessments will be as clinically indicated.

## Post Treatment Assessments (post-treatment weeks 1, 12 and 36)

All enrolled subjects will complete the post-treatment weeks 1, 12 and 36 visits, regardless of treatment duration. The post-treatment visits will be scheduled from the date of last administration of study drug, with the exception of the post-treatment week 1 visit, which will be 1 week after week 8 or the ET visit.

Post-treatment week 1 visit will include review of adverse events, provision of end-of-treatment HCV RNA results, and motivational interviewing–based risk reduction counseling.

Post-treatment week 12 and 36 visits will include review of adverse events, urine drug screen, ACASI, HCV RNA, stored samples for potential testing of SOF and LDV metabolite levels, and HCV RNA sequencing.

Subjects will be compensated $15 for post-treatment week 1 visit and $50 for each post-treatment weeks 12 and 36 visits.

## Audio Computer Assisted Self Interview (ACASI)

The ACASI, which is administered at enrollment and weeks 4, 8, and post-treatment weeks 12 and 36, will include injection drug use and injection risk behavior, partner-by-partner data on injection/substance use/HCV status, demographic information (enrollment only), drug use history (enrollment only), quality of life scale (SF-12), Center for Epidemiologic Studies Depression Rating Scale, and Severity of Dependence Scale and participant satisfaction with treatment (week 8 only).

## Assessments for Premature Discontinuation from Study

Discontinuation from study drug dosing and discontinuation from the overall study including follow-up will be collected as two separate events. If a subject discontinues study treatment dosing, every attempt will be made to keep the subject in the study and continue to perform an early termination visit (see Section 6.4) and the required study-related follow-up and procedures (see Section 6.6). This includes post-treatment weeks 1, 12 and 36 visits. If this is not possible or acceptable to the subject or investigator, the subject may be withdrawn from the study.

## End of Study

Subjects are considered to have completed the study after the post-treatment week 36 follow-up visit, regardless of treatment duration and early termination from study drug.

## Procedures and Specifications

### Clinical Laboratory Analytes

Hematology: White Blood Cells (WBC), Red Blood Cells (RBC), Hemoglobin, Hematocrit, MCV, MCH, HCHC, Platelets, with differential, percentages and absolute values, Neutrophils, Lymphocytes, Monocytes, Eosinophils, Basophils, Large Lymphocytes.

Coagulation: Prothrombin time (PT), INR.

Chemistry: Sodium, Potassium, Chloride, Carbon Dioxide, Anion Gap, BUN, Creatinine, eGFR, eGFR African American, Glucose, Total Bilirubin, Total Protein, Albumin, Calcium, Alkaline Phosphatase, AST, ALT.

Virological Tests: Serologies for HCV RNA will be measured using the COBAS^®^ AmpliPrep/COBAS^®^ TaqMan^®^ HCV Quantitative Test, version 2.0. HCV genotype and subtype will be determined using the Siemens VERSANT^®^ HCV Genotype INNO-LiPA 2.0 Assay.

Pregnancy Test: Urine β-hCG

Urine Toxicology: 11 Panel Muti-Drug (Cocaine, Amphetamine, Methamphetamine, THC, Methadone, Opiates, Oxycodone, PCP, Barbituates, Benzodiazepines, and Buprenorphine) Screen Test Card using lateral flow chromatographic immunoassay for the qualitative detection of drugs and drug metabolites at cut-off concentrations that follow guidelines set by the Substance Abuse and Mental Health Services Administration (SAMHSA).

### Medical History

Medical history including details regarding illnesses and allergies, date(s) of onset, and whether condition(s) is currently ongoing, and medication history will be collected on all subjects during screening.

### Complete Physical Examination

A complete physical examination will include source documentation of general appearance, and the following body systems: Head, neck, eyes, ears, nose, throat, mouth and tongue; respiratory; cardiovascular; lymph nodes; abdomen; skin, hair, nails; musculoskeletal; neurological.

### Vital Signs

Vital sign collection will include measurement of resting blood pressure, pulse, respiratory rate, and temperature.

Blood pressure will be measured using the following standardized process:

- Subject will sit with feet flat on the floor and measurement arm supported so that the midpoint of the manometer cuff is at heart level;
- Use a mercury sphygmomanometer or automatic blood pressure device with an appropriately sized cuff with the bladder centered over the brachial artery;
- Measure and record the blood pressure to the nearest 2 mm Hg mark on the manometer or to the nearest whole number on an automatic device.

### Creatinine Clearance

Creatinine clearance is calculated by the Cockcroft-Gault equation [^12^](#_ENREF_12) using actual body weight (BW).

Male: CL_cr_ (mL/min) = [140 - age (years)] × BW(kg)
 72 × S_cr_

Female: CL_cr_ (mL/min) = [140 - age (years)] × BW(kg) × 0.85
 72 × S_cr_

S_cr_ = serum creatinine (mg/dL)

### Body Mass Index (BMI)

BMI is calculated by the following equation.

BMI = weight (pounds) × 703 or weight in kilograms
 (height in inches)^2^ (height in meters)^2^

### The Fibrosis-4 Score (FIB-4):

FIB-4 is calculated by the following equation.

FIB-4 = Age (years) x AST (U/L)

Platelet Count (10 9/L) x √ ALT (U/L)

If FIB-4 <1.45, no or minimal fibrosis, If FIB-4 >3.25, significant fibrosis.

**Excluded from study if FIB-4 >3.25.**

### Fibrosis-Cirrhosis Index (FCI):

FCI is calculated by the following equation.

### FCI = ALP × Bilirubin

### Albumin × Platelet count

### Excluded from study if FCI >1.25.

### Viral RNA Sequencing / Phenotyping Sample

Plasma samples will be collected at screening, weeks 2 and 8, and post-treatment weeks 12 and 36 for potential future HCV viral sequence analysis.

Details regarding the collection, processing, and shipping of samples will be included in the lab manual.

### Pharmacokinetic (PK) Samples

Blood samples will be collected and stored for potential PK testing for all subjects at weeks 2, 4 and 8. PK analysis of SOF (and its metabolites GS‑566500 and GS‑331007) and LDV may be conducted.

Details regarding the collection, processing, and shipping of samples will be included in the lab manual.

### Dried Blood Spot Collection

Dried blood spots will be collected at weekly visits 1, 2, 3, 4, 5, 6, 7, 8 post-treatment weeks 12 and 36, and at the early termination visit (if applicable). The dried blood spots will be stored for potential future testing of SOF and LDV metabolite levels and HCV RNA.

Details regarding the collection, processing and shipment of samples will be included in the lab manual.

### Pregnancy Testing

All females of childbearing potential will have urine pregnancy testing at enrollment. Following enrollment a self-reported positive pregnancy test will be confirmed by urine testing or documentation and the two physicians on the study will review the case to determine if treatment is to be discontinued or continued. This will be decided based on clinical review, incorporating at least the following characteristics: duration of treatment remaining, plans for continuing or terminating pregnancy and severity of liver disease.

### Quality of Life Surveys

The quality of life scale included in this study is the EQ5-D. It will be completed by patients at enrollment, weeks 4 and 8, post-treatment weeks 12 and 36, and early termination (if applicable) regardless of study arm. The Quality of Life Survey will be included in the ACASI.

### Depression Rating Scale

The depression rating scale included in this study is the Center for Epidemiologic Studies Depression Rating Scale. It will be completed by patients at enrollment, weeks 4 and 8, post-treatment weeks 12 and 36 and early termination (if applicable) regardless of study arm. The Depression Rating Scale will be included in the ACASI.

### Severity of Dependence Scale

The Severity of Dependence Scale will be completed by patients at enrollment, weeks 4 and 8, post-treatment weeks 12 and 36 and early termination (if applicable) regardless of study arm. The Severity of Dependence Scale will be included in the ACASI.

# TOXICITY MANAGEMENT

## Subject Stopping Rules

Due to a clinical or laboratory event, administration of study drug may be discontinued. There is no option for LDV/SOF dose reduction. If LDV/SOF is stopped due to toxicity, it must not be restarted, and the subject will complete an ET visit. Post-treatment week 1, 12 and 36 visits will also be scheduled.

Subjects who meet any of the following laboratory criteria must stop study drug:

- Elevation of ALT and/or AST >5x Day 1 or nadir
- Abnormal elevation of ALT >3 x Day 1
- Elevation of ALT >15 x ULN
- Any Grade 3 or greater rash associated with constitutional symptoms
- Any Grade 4 adverse event or laboratory abnormality assessed as related to LDV/SOF

# ADVERSE EVENTS MANAGEMENT

## Definition of Adverse Events, Adverse Reactions, and Serious Adverse Events

### Adverse Event

An adverse event (AE) is any untoward medical occurrence in a clinical study subject administered a pharmaceutical medicinal product, which does not necessarily have a causal relationship with the treatment. An AE can therefore be any unfavorable and/or unintended sign, symptom, or disease temporally associated with the use of a medicinal product, whether or not considered related to the medicinal product. AEs may also include pre- or post‑treatment complications that occur as a result of protocol specified procedures, lack of efficacy, overdose, substance use, or occupational exposure. Preexisting events that increase in severity or change in nature during or as a consequence of participation in the clinical study will also be considered AEs.

An expected adverse event is an AE that may be reasonably anticipated to occur as a result of the study procedures or study participation or is part of the normal disease process or progression.

An unexpected adverse event is defined as being unexpected if the event exceeds the nature, severity, or frequency described in the current Committee on Human Research (CHR) application including the protocol, consent form and investigator brochure, when applicable. An unexpected AE also includes any AE that meets the following criteria:

- Results in subject withdrawal from study participation
- Due to an overdose of study medication
- Due to a deviation from CHR-approved study protocol

An AE does not include the following:

- Medical or surgical procedures (e.g., surgery, endoscopy, tooth extraction, transfusion) performed; the condition that leads to the procedure may be an adverse event and must be reported.
- Pre-existing diseases or conditions or laboratory abnormalities present or detected during or before screening that do not worsen
- Situations where an untoward medical occurrence has not occurred (e.g., hospitalization for elective surgery, social and/or convenience admissions)
- Overdose without clinical sequelae (see Section 8.5)
- Any medical condition or clinically significant laboratory abnormality with an onset date before enrollment and not related to a protocol-associated procedure is not an AE. It is considered to be pre-existing and should be documented on the medical history.
- Substance use or admission to substance use treatment
- Incarceration

### Serious Adverse Events

A **serious adverse event** (SAE) is defined as an event that results in the following:

- Death
- Life-threatening event (Note: The term “life-threatening” in the definition of “serious” refers to an event in which the subject was at risk of death at the time of the event; it does not refer to an event that hypothetically might have caused death if it were more severe.)
- In-patient hospitalization or prolongation of existing hospitalization
- Persistent or significant disability/incapacity
- A congenital anomaly/birth defect or cancer
- A medically important event or reaction: such events may not be immediately life-‑threatening or result in death or hospitalization but may jeopardize the subject or may require intervention to prevent one of the other outcomes constituting SAEs. Medical and scientific judgment must be exercised to determine whether such an event is reportable under expedited reporting rules. Examples of medically important events include intensive treatment in an emergency room or at home for allergic bronchospasm, blood dyscrasias or convulsions that do not result in hospitalization. For the avoidance of doubt, infections resulting from contaminated medicinal product will be considered a medically important event and subject to expedited reporting requirements.
- Event that changes the risk/benefit ratio of the study

### Clinical Laboratory Abnormalities and Other Abnormal Assessments as Adverse Events or Serious Adverse Events

Laboratory abnormalities without clinical significance are not recorded as AEs or SAEs. However, laboratory abnormalities (e.g., clinical chemistry, hematology, and urinalysis) that require medical or surgical intervention or lead to investigational medical product (IMP) interruption, modification, or discontinuation must be recorded as an AE, as well as an SAE, if applicable. In addition, laboratory or other abnormal assessments that are associated with signs and/or symptoms will be recorded as an AE or SAE if they meet the definition of an AE or SAE as described in Sections 8.1.1 and 8.1.2. If the laboratory abnormality is part of a syndrome, record the syndrome or diagnosis (e.g., anemia), not the laboratory result (i.e., decreased hemoglobin).

## Assessment of Adverse Events and Serious Adverse Events

The investigator or qualified sub-investigator is responsible for assessing AEs and SAEs for causality and severity, and for final review and confirmation of accuracy of event information and assessments.

### Assessment of Causality for Study Drug and Procedures

The investigator or qualified sub-investigator is responsible for assessing the relationship to study procedures or medications using clinical judgment and the following considerations:

- **Definitely not**: Evidence exists that the adverse event has an etiology other than the IMP. For SAEs, an alternative causality must be provided (e.g., pre-existing condition, underlying disease, intercurrent illness, or concomitant medication).
- **Probably not**: Adverse event has improbable time relationship to intake of IMP, and attribution with disease or other drugs is likely.
- **Possibly**: Adverse event has reasonable time relationship to intake of IMP, and attribution with disease or other drugs is possible.
- **Probably**: Adverse event has reasonable time relationship to intake of IMP and attribution with disease or other drugs is unlikely.
- **Definitely**: There is reasonable probability that the event may have been caused by the investigational medicinal product.

Ineffective treatment should not be considered as causally related in the context of AE reporting.

The relationship to study procedures (e.g., invasive procedures such as venipuncture) should be assessed using the following considerations:

- **Definitely not**: Evidence exists that the adverse event has an etiology other than the study procedure. For SAEs, an alternative causality must be provided (e.g., pre-existing condition, underlying disease, intercurrent illness, or concomitant medication).
- **Probably not**: Adverse event has improbable time relationship to intake of study procedure, and attribution with disease or other drugs is probable.
- **Possibly**: Adverse event has reasonable time relationship to intake of study procedure and attribution with disease or other drugs is possible.
- **Probably**: Adverse event has reasonable time relationship to intake of study procedure and attribution with disease or other drugs is unlikely.
- **Definitely**: There is reasonable probability that the event may have been caused by the study procedures.

## Assessment of Severity

Severity should be recorded and graded according to the Division of AIDS Table for Grading the Severity of Adult and Pediatric Adverse Events (DAIDS) Version 2.0 November 2014.

## Investigator Requirements for Reporting Adverse Events and Serious Adverse Events

### Reporting to UCSF Committee on Human Research (CHR / IRB)

All SAEs and unexpected AEs of relation greater than unrelated must be reported within 5-working-days of PI awareness using the Adverse Event Reporting Form on the UCSF CHR website (iRIS).

### Reporting to UCLA

Reporting to UCLA Data Safety Monitoring Board (DSMB)

All SAEs and unexpected AEs meeting reportable criteria in the Data Safety Monitoring Plan (DSMP) must be reported within 10-working-days of PI awareness. A summary letter must include detailed accounts of the following:

- Summary of SAE
- Resolution
- Study Drug
- Attribution
- Notification

### Reporting to NIH – National Institute of Drug Abuse (NIDA) through the Serious Events Tracking and Reporting System (SAETRS)

All SAEs meeting reportable criteria in the DSMP must be reported within 10-working-days of PI awareness. If SAE is fatal, it must be reported within 2-working-days of PI awareness.

### Investigator Requirements and Instructions for Reporting Adverse Events and Serious Adverse Events to Gilead

*Adverse Events*

The investigator shall report SAEs that occur during treatment and within 30 days of last dose of study drug to Gilead quarterly in the format of a Study Progress Report.

*Serious Adverse Events*

All SAEs, regardless of cause or relationship, that occur from enrollment until 30 days after the last dose of study drug shall be reported to Gilead Sciences DSPH within 10 working days of PI awareness. If SAE is fatal, it shall be reported within 2 working days of PI awareness. If the investigator learns of any SAEs that occur after study participation has concluded and the event is deemed relevant to the use of study drug, he will promptly document and report the event to Gilead Sciences DSPH.

**Gilead Sciences DSPH**

Fax: +1-650-522-5477

E-mail: Safety_FC@gilead.com

## Special Situations Reports

### Definitions of Special Situations

Special situation reports include all reports of medication error, abuse, misuse, overdose, lack of effect reports and pregnancy reports regardless of an associated AE.

Medication error is any unintentional error in the prescribing, dispensing, or administration of a medicinal product while in the control of the health care provider, subject, or consumer.

Abuse is defined as persistent or sporadic intentional excessive use of a medicinal product by a subject.

Misuse is defined as any intentional and inappropriate use of a medicinal product that is not in accordance with the protocol instructions or the local prescribing information.

An overdose is defined as an accidental or intentional administration of a quantity of a medicinal product given per administration or cumulatively which is above the maximum recommended dose as per protocol or in the product labelling (as it applies to the daily dose of the subject in question). In cases of a discrepancy in drug accountability, overdose will be established only when it is clear that the subject has taken the excess dose(s). Overdose cannot be established when the subject cannot account for the discrepancy except in cases in which the investigator has reason to suspect that the subject has taken the additional dose(s).

Product complaint is defined as complaints arising from potential deviations in the manufacture, packaging, or distribution of the medicinal product.

### Instructions for Reporting Special Situations

#### **Instructions for Reporting Pregnancies**

The investigator should report pregnancies in female study subjects that are identified after initiation of study drug and throughout the study, including the post study drug follow-up‑ period, to Gilead Sciences DSPH using the pregnancy report form within 10 working days of becoming aware of the pregnancy.

The pregnancy itself is not considered an AE nor is an induced elective abortion to terminate a pregnancy without medical reasons.

Any premature termination of pregnancy (eg, a spontaneous abortion, an induced therapeutic abortion due to complications or other medical reasons) will be reported within 10 working days as an SAE. The underlying medical reason for this procedure should be recorded as the AE term.

A spontaneous abortion is always considered to be an SAE and will be reported as described in Sections 8.4.4. Furthermore, any SAE occurring as an adverse pregnancy outcome post study will be reported to Gilead Sciences DSPH.

The outcome of the pregnancy should be reported to Gilead Sciences DSPH using the pregnancy outcome report form.

#### **Reporting Other Special Situations**

All other special situations will be reported as AEs or SAEs and reported accordingly. These reports must consist of situations that involve study drug but do not apply to non-Gilead concomitant medications.

# STATISTICAL CONSIDERATIONS

## Analysis Objectives and Endpoints

### Analysis Objectives

The primary objectives of this study are:

- To assess the feasibility of treating active PWIDs for HCV with LDV/SOF by mDOT versus weekly dispensing, with MI adherence counseling, as measured by recruitment and retention rates, overall and by arm.
- To evaluate the acceptability of mDOT versus weekly dispensing, with MI adherence support, among PWIDs treated with LDV/SOF, as determined by mDOT and MI participation rates, medication adherence rates and the proportion of subjects that complete therapy, by arm.
- To assess through in-depth, semi-structured qualitative interviews, the challenges with time intensity required for mDOT and weekly dispensing interventions, and identify key factors affecting treatment adherence for PWIDs treated with LDV/SOF.

The exploratory objectives of this study are:

- To assess end-of-treatment response and SVR, as determined by the proportion with undetectable HCV RNA at weeks 8 and post treatment week 12, by arm.
- To assess adherence to treatment by weekly SOF metabolite levels, by arm, If SVR, retention and/or medication adherence data suggestion medication compliance issues.
- To assess drug use behaviors before and after HCV treatment.
- To assess HCV relapse and reinfection at post treatment week 36 among participants achieving SVR.
- To evaluate demographic, behavioral and HCV strain correlates of adherence and SVR.
- To characterize the social and PWID networks of participants in preparation for a subsequent full TasP trial.

### Primary Endpoints

- Screen to eligibility ratio, overall
- Eligibility to enrollment rate, overall
- Retention rates, overall and by arm
- Study completion rates, overall and by arm
- Medication adherence rates and the proportion of subjects that complete therapy, overall and by arm
- Barriers and facilitators to HCV treatment

### Secondary Endpoints

Secondary endpoints include the following:

- SVR, as determined by the proportion with undetectable HCV RNA at weeks 8 and post treatment week 12, by arm
- SOF metabolite levels, by arm, as needed based on results.
- HCV risk behaviors and substance use before and after HCV treatment
- HCV relapse and reinfection at post treatment week 36 among participants achieving SVR.

### Other Endpoints of Interest

Additional evaluations will explore demographic, behavioral and HCV strain correlates of adherence and SVR; and analyses exploring at the social and PWID networks of participants in preparation for a subsequent full TasP trial.

## Analysis Conventions

All individual subject data will be listed as measured. All statistical summaries and analyses will be performed using STATA^®^ software. The study drug in this study is LDV/SOF. Last dose of study drug refers to the last dose of LDV/SOF and will be used in the definition of treatment-emergent AEs and laboratory abnormalities as well as the efficacy endpoints of SVR at various post-treatment time points. Between group analyses will be conducted using intention-to-treat analyses without regard to study procedure compliance.

## Demographic Data and Baseline Characteristics

Demographic and baseline characteristics will be summarized using standard descriptive methods by treatment group and overall.

Demographic data will include sex, self-identified race/ethnicity, and age.

Baseline characteristic data will include body mass index, HCV RNA level (log_10_ IU/mL) and additional endpoints as necessary.

## Data Analysis

### Primary Analysis

### Feasibility: Feasibility of treating active PWIDs for HCV with LDV/SOF by mDOT versus unobserved dosing will be measured by recruitment and retention rates overall and by arm. We will calculate the following feasibility measures: 1) proportion of those eligible among those screened; 2) proportion enrolled among those eligible; and 3) study retention and completion rates, overall and by arm. Process measures (e.g., visit length) and reactions to study procedures will also be assessed. Descriptive statistics will be presented with 95% confidence intervals, to help in interpretation. Between-group differences in retention and completion will be assessed using Fisher’s exact and Wilcoxon tests. We will also calculate Kaplan-Meier curves for time to dropout, by group, and test for differences using the log-rank test.

**Acceptability:** The acceptability of mDOT versus unobserved dosing, the percent of treatment medication adherence to LDV/SOF, will be measured by the percent of doses taken overall (observed and unobserved), will be assessed using DOT doses and weekend WisePill data for the mDOT arm, and WisePill data for the unobserved dosing arm. Other outcomes of interest will include completion of therapy, patterns of adherence, and time to stopping medication. Concordance of the various adherence measures will be examined using weighted Kappa and intraclass correlations. Between-arm differences will be assessed using Fisher’s exact and Wilcoxon tests for outcomes measured once, and GEE models for repeated outcomes. We will also use GEE models to compare WisePill openings and pill counts, accounting for between-arm differences in expected frequency.

**Challenges from mDOT:** In-depth, semi-structured qualitative interviews will assess the challenges with time intensity required for mDOT versus unobserved dosing, and identify key factors affecting treatment adherence for PWIDs treated with LDV/SOF. Transcribed semi-structured interviews will be imported into Atlas.ti, a qualitative research software program. We will utilize two qualitative analytic methods to interpret data. First, content analysis will allow us to focus on answering study questions regarding experiences with LDV/SOF treatment and DOT strategies, and opinions on challenges to achieving consistent adherence to treatment. A priori themes of interest primarily focused on the time-intensity required for this treatment plan and based upon the team’s experience with HCV treatment among PWIDs will be developed and used as the first codebook with which data are coded. Second, thematic analysis with open coding will allow themes to emerge from the data. A coding system will be developed using an iterative process. In the initial phase, Dr.s Wilson and Matheson will review all transcripts and field notes to become familiar with the data, and the analytic team will make notes of their observations and potential codes for use in the initial analysis meeting. Then a codebook, consisting of the code definition and inclusion and exclusion criteria, will be created to aid analysis. In addition to predetermined HCV treatment dimensions, additional codes will be developed independently by each analytic team member through an inductive process of identifying themes that emerge from the data. Group analysis meetings will be held to compare independently developed codes for similarity and further definition. A consensus will be reached regarding each code, its application and definition. To ensure consistency, a codebook and dictionary will be developed with universal definitions for each code.

Inter-rater reliability: To ensure reliability and validity in open-ended component of the in-depth interviews analysis, we will implement a verification strategy to ensure self-correction throughout the analysis process. The two coders will assess inter-rater reliability by calculating the correlation between a set of ratings done by two independent raters for the initial set of 3-6 transcripts, and then again on the next set of 6 transcripts. After each inter-rater reliability assessment, the codebook and codes will be revised based on reconciliation of findings done between coders. Inter-rater reliability will be assessed as a team twice during the analysis process and again at the end of analysis as a post-hoc evaluation of reliability and validity. This evolving analysis process will ensure high quality of research decisions, the rationale behind decisions, and the responsiveness of investigators to the data. Once the codebook is developed and verified all remaining transcripts will be coded. Further meetings will be held to discuss any differences in coding and to ensure consistency in the application of codes.

Final analysis will begin with data reduction, then display, analysis and conclusion drawing using a conceptual framework in which hypothesized factors affecting adherence are assessed and unanticipated findings are identified. The analytic team will work from the coded data to merge findings into a final summary and a consensus of major themes, relationships between themes, and ranking of items of most importance to adherence. This method of data reduction encompassing both site and team based analysis creates a robust iterative process through which the data is thoroughly discussed and analytical consensus achieved. Atlas.ti software will be used to both code and develop data displays used in the analysis for this study.

### Exploratory Analyses

While we recognize the small sample size limits our ability to draw firm conclusions from exploratory analyses, the paucity of data for this treatment approach allows for hypothesis-generating results.

End-of-treatment response and SVR: We will compare the proportion of participants with undetectable HCV RNA at weeks 8 and post-treatment week 12 between arms using Fisher’s exact tests.

Acceptability based on drug levels: Depending on resource availability and SVR rates, we may calculate rates of LDV and/or SOF metabolite-positivity by week in both arms; between-group differences will be compared using GEE models for repeated outcomes.

HCV relapse and reinfection: Among participants who achieve SVR, we will determine the proportion who experience HCV relapse and reinfection at post-treatment week 36, overall and by arm, with exact 95% confidence intervals.

Social and PWIDs network analyses to inform a subsequent full TasP trial: We will characterize injector network sizes at baseline and follow-up. Newman’s method will be used to calculate assortativity coefficients, a measure of the degree of demographic and risk behavior similarity within networks. These findings will help us determine whether a full efficacy trial should use targeted sampling strategies to ensure diversity in network sizes, and whether randomization should be stratified by one or both of these factors in future trials.

Other Exploratory Outcomes:

Drug use behaviors before and after HCV treatment: Recent data suggest significant changes in drug use behaviors after HCV diagnosis: serosorting with other HCV-positive PWIDs and a sustained reduction in drug use. We will assess these behaviors at baseline and follow-up for preliminary estimates of the possible impact of HCV treatment on these behaviors. ANCOVA and conditional logistic models will be used for continuous and binary outcomes respectively.

Correlates of Adherence and SVR: We will explore demographic, behavioral correlates of medication adherence using GEE models for repeated measures. In addition, we will explore demographic and behavioral correlates of SVR using Fisher exact tests and exact logistic regression.

## Sample Size

**Sample size justification:** The sample of 30 will provide typical precision for a pilot study of feasibility and acceptability outcomes. Specifically, we will be able to estimate the mean of continuous outcomes within margins of sampling error (MSEs; half-widths of 95% confidence intervals) of 0.37 standard deviations (SD) overall, and 0.55 SDs within arm; percentages will be estimated within MSEs of 11-18 points overall, and 15-25 points within arm, depending on the sample value. In designing the full-scale trial, these exploratory results will be cautiously interpreted in the light of confidence intervals, plausibility, and prior results.

# RESPONSIBILITIES

## Investigator Responsibilities

### Good Clinical Practice

The investigator will ensure that this study is conducted in accordance with the principles of the Declaration of Helsinki (as amended in Edinburgh, Tokyo, Venice, Hong Kong, and South Africa), International Conference on Harmonisation (ICH) guidelines, or with the laws and regulations of the country in which the research is conducted, whichever affords the greater protection to the study subject.

The investigator and all applicable subinvestigators will comply with 21 CFR, Part 54, 1998, providing documentation of their financial interest or arrangements with Gilead, or proprietary interests in the investigational drug under study. This documentation must be provided prior to the investigator’s (and any subinvestigator’s) participation in the study. The investigator and subinvestigator agree to notify Gilead of any change in reportable interests during the study and for 1 year following completion of the study. Study completion is defined as the date when the last subject completes the protocol-defined activities.

### Institutional Review Board (IRB) Approval

The investigator (or sponsor as appropriate according to local regulations) will submit this protocol, informed consent form, and any accompanying material to be provided to the subject (such as advertisements, subject information sheets, or descriptions of the study used to obtain informed consent) to an IRB. The investigator will not begin any study subject activities until approval from the IRB has been documented and provided as a letter to the investigator.

Before implementation, the investigator will submit to and receive documented approval from the IRB any modifications made to the protocol or any accompanying material to be provided to the subject after initial IRB approval, with the exception of those necessary to reduce immediate risk to study subjects.

### Informed Consent

Study staff are responsible for obtaining written informed consent from each individual participating in this study after adequate explanation of the aims, methods, objectives, and potential hazards of the study and before undertaking any study-related procedures. The investigator will use the most current IRB approved consent form for documenting written informed consent. Each informed consent will be appropriately signed and dated by the subject or the subject’s legally authorized representative and the person conducting the consent discussion, and also by an impartial witness if required by IRB or by local requirements. The consent form will inform subjects about sample retention and use of retained samples.

### Confidentiality

The investigator will assure that subjects’ anonymity will be strictly maintained and that their identities are protected from unauthorized parties. Only subject initials, date of birth, and a unique identifier (as allowed by local law) will be recorded on any form or biological sample submitted to the Sponsor, IRB, or laboratory. Laboratory specimens will be labeled in such a way as to protect subject identity while allowing the results to be recorded to the proper subject. Refer to specific laboratory instructions. NOTE: If given signed permission by the subject the investigators keeps a locator form showing names, date of birth and addresses for all subjects screened and for all subjects enrolled in the trial. Subject data will be processed in accordance with all applicable regulations.

### Study Files and Retention of Records

The investigator will maintain adequate and accurate records to enable the conduct of the study to be fully documented and the study data to be subsequently verified. These documents should be classified into at least the following two categories: (1) investigator’s study file, and (2) subject clinical source documents.

The investigator’s study file will contain the protocol/amendments, CRF and query forms, IRB and governmental approval with correspondence, informed consent, drug records, staff curriculum vitae and authorization forms, and other appropriate documents and correspondence.

The required source data should include sequential notes containing at least the following information for each subject:

- Subject identification (name, date of birth, gender);
- Documentation that subject meets eligibility criteria, ie, history, physical examination, and confirmation of diagnosis (to support inclusion and exclusion criteria);
- Documentation of the reason(s) a consented subject is not enrolled
- Participation in study (including study number);
- Study discussed and date of informed consent;
- Dates of all visits;
- Documentation that protocol specific procedures were performed;
- Results of efficacy parameters, as required by the protocol;
- Start and end date (including dose regimen) of study drug, including dates of dispensing and return;
- Record of all adverse events and other safety parameters (start and end date, and including causality and severity);
- Concomitant medication (including start and end date, dose if relevant; dose changes);
- Date of study completion and reason for early discontinuation, if it occurs.

### Case Report Forms

For each subject consented, a CRF will be completed by an authorized study staff member whose training for this function is documented according to study procedures. CRF should be completed on the day of the subject visit to enable the sponsor to perform central monitoring of safety data. Prior to database lock (or any interim time points as described in the clinical data management plan), study staff will use his/her log in credentials to confirm that the forms have been reviewed, and that the entries accurately reflect the information in the source documents. The CRF capture the data required per the protocol schedule of events and procedures.

### Investigational Medicinal Product Accountability and Return

Used (empty or partially empty) and unused study drug supplies will be destroyed on site according to appropriate standard operating procedure (SOP). A copy of the site’s approved SOP will be obtained for central files.

If study drug is destroyed on site, the investigator must maintain accurate records for all study drug destroyed. Records must show the identification and quantity of each unit destroyed, the method of destruction, and the person who disposed of the study drug. Upon study completion, copies of the study drug accountability records must be filed at the site. Another copy will be returned to Gilead.

The study monitor will review study drug supplies and associated records at periodic intervals.

### Inspections

The investigator will make available all source documents and other records for this trial to Gilead’s appointed study monitors, to IRBs, or to regulatory authority or health authority inspectors.

### Protocol Compliance

The investigator is responsible for ensuring the study is conducted in accordance with the procedures and evaluations described in this protocol.

## Gilead Responsibilities

### Protocol Modifications

Protocol modifications, except those intended to reduce immediate risk to study subjects, may be made by the investigator. The investigator must submit all protocol modifications to the IRB in accordance with local requirements and receive documented IRB approval before modifications can be implemented. The investigator will also notify Gilead.

### Study Report and Publications

No such communication, presentation, or publication will include Gilead’s confidential information (see Section 10.1.4).

The investigator will comply with Gilead’s request to delete references to its confidential information (other than the study results) in any paper or presentation.

## Joint Investigator/Gilead Responsibilities

### Payment Reporting

Investigators and their study staff may be asked to provide services performed under this protocol, e.g. attendance at Investigator's Meetings. If required under the applicable statutory and regulatory requirements, Gilead will capture and disclose to Federal and State agencies any expenses paid or reimbursed for such services, including any clinical trial payments, meal, travel expenses or reimbursements, consulting fees, and any other transfer of value.

### Access to Information for Monitoring

In accordance with regulations and guidelines, the study monitor must have direct access to the investigator’s source documentation in order to verify the accuracy of the data recorded in the CRF.

### Access to Information for Auditing or Inspections

Representatives of regulatory authorities or of Gilead may conduct inspections or audits of the clinical study. The investigator agrees to provide to representatives of a regulatory agency or Gilead access to records, facilities, and personnel for the effective conduct of any inspection or audit.

### Study Discontinuation

The investigator reserves the right to terminate the study at any time. Should this be necessary, both the investigator and Gilead will arrange discontinuation procedures and notify the appropriate regulatory authority(ies) and IRB. In terminating the study, Gilead and the investigator will assure that adequate consideration is given to the protection of the subjects’ interests.

# REFERENCES

**1.** Negro F, Alberti A. The global health burden of hepatitis C virus infection. *Liver international : official journal of the International Association for the Study of the Liver.* Jul 2011;31 Suppl 2:1-3.

**2.** Coffin PO, Jin H, Huriaux E, Mirzazadeh A, Raymond HF. Trends in use of health care and HIV prevention services for persons who inject drugs in San Francisco: results from National HIV Behavioral Surveillance 2005-2012. *Drug Alcohol Depend.* Jan 1 2015;146:45-51.

**3.** Zibbell JE, Iqbal K, Patel RC, et al. Increases in hepatitis C virus infection related to injection drug use among persons aged </=30 years - kentucky, tennessee, virginia, and west virginia, 2006-2012. *MMWR Morb Mortal Wkly Rep.* May 8 2015;64(17):453-458.

**4.** Page K, Hahn JA, Evans J, et al. Acute hepatitis C virus infection in young adult injection drug users: a prospective study of incident infection, resolution, and reinfection. *The Journal of infectious diseases.* Oct 15 2009;200(8):1216-1226.

**5.** Martin NK, Vickerman P, Grebely J, et al. Hepatitis C virus treatment for prevention among people who inject drugs: Modeling treatment scale-up in the age of direct-acting antivirals. *Hepatology.* Mar 28 2013.

**6.** Page K, Morris MD, Hahn JA, Maher L, Prins M. Injection drug use and hepatitis C virus infection in young adult injectors: using evidence to inform comprehensive prevention. *Clin Infect Dis.* Aug 2013;57 Suppl 2:S32-38.

**7.** Page K, Osburn W, Evans J, et al. Frequent longitudinal sampling of hepatitis C virus infection in injection drug users reveals intermittently detectable viremia and reinfection. *Clin Infect Dis.* Feb 2013;56(3):405-413.

**8.** Hilsden RJ, Macphail G, Grebely J, Conway B, Lee SS. Directly observed pegylated interferon plus self-administered ribavirin for the treatment of hepatitis C virus infection in people actively using drugs: a randomized controlled trial. *Clin Infect Dis.* Aug 2013;57 Suppl 2:S90-96.

**9.** Cousien A, Tran VC, Jauffret-Roustide M, Deuffic-Burban S, Dhersin JS, Yazdanpanah Y. Impact of new DAA-containing regimens on HCV transmission among injecting drug users (IDUs): a model-based analysis (#12376). *49th International Liver Congress*. London, UK2014.

**10.** Krook AL, Stokka D, Heger B, Nygaard E. Hepatitis C treatment of opioid dependants receiving maintenance treatment: results of a Norwegian pilot study. *Eur Addict Res.* 2007;13(4):216-221.

**11.** Waizmann M, Ackermann G. High rates of sustained virological response in hepatitis C virus-infected injection drug users receiving directly observed therapy with peginterferon alpha-2a (40KD) (PEGASYS) and once-daily ribavirin. *J Subst Abuse Treat.* Jun 2010;38(4):338-345.

**12.** Cockcroft DW, Gault MH. Prediction of creatinine clearance from serum creatinine. *Nephron.* 1976;16:31-41.

**APPENDICES**

Appendix 1. Study Procedures Table

Appendix 2. Estimating Severity Grade for Parameters Not Identified in the Grading Table

Appendix 1. Study Procedures Table

Screening, Weekly Visits (mDOT and unobserved dosing arms), and Post-Treatment Visits

| Clinical Assessments | | Screening visits | Enrollment | Visit identified by study week | | | | | | | | | | Post-treatment visits | | | |
| --- | --- | --- | --- | --- | --- | --- | --- | --- | --- | --- | --- | --- | --- | --- | --- | --- | --- |
|  |  | Day ‑30^a^to Day 0 |  | 1 | 2 | 3 | | 4 | 5 | 6 | 7 | 8 | 1 | | 12 | 36 | ET |
| Informed Consent | | X* |  |  |  |  |  | |  |  |  |  |  | |  |  |  |
| Determine Eligibility | | X* |  |  |  |  |  | |  |  |  |  |  | |  |  |  |
| Medical History ^b^ | | X |  |  |  |  |  | |  |  |  |  |  | |  |  |  |
| Physical Examination | | X |  |  |  |  | X | |  |  |  | X |  | |  |  | X |
| Review of systems | | X |  |  |  |  |  | |  |  |  |  |  | |  |  |  |
| Height and weight | | X |  |  |  |  |  | |  |  |  |  |  | |  |  |  |
| Vital Signs ^c^ | | X | X |  |  |  | X | |  |  |  | X |  | |  |  | X |
| Concomitant Medications | | X |  | X | X | X | X | | X | X | X |  |  | |  |  | X |
| Adverse Events | |  |  | X | X | X | X | | X | X | X | X | X | | X | X | X |
| PT/INR | | X |  |  |  |  |  | |  |  |  |  |  | |  |  |  |
| CBC with diff/CMP | | X |  |  | X |  |  | |  |  |  | X |  | |  |  | X |
| HCV RNA (Plasma) | | X |  |  | X |  |  | |  |  |  | X |  | | X | X | X |
| Urine β‑hCG ^d^ | |  | X |  |  |  |  | |  |  |  |  |  | |  |  |  |
| Urine Drug Screen | | X* | X | X | X | X | X | | X | X | X | X | X | | X | X | X |
| HCV Genotyping^e^ | | X |  |  |  |  |  | |  |  |  |  |  | |  |  |  |
| HIV Ag/Ab | | X |  |  |  |  |  | |  |  |  | X |  | |  |  | X |
| HBsAg | | X |  |  |  |  |  | |  |  |  |  |  | |  |  |  |
| Stored dried blood spots | |  |  | X | X | X | X | | X | X | X | X |  | | X | X | X |
| Stored samples for SOF and LDV metabolite level | |  |  |  | X |  | X | |  |  |  | X |  | |  |  | X |
| Stored samples for HCV sequence analysis | | X |  |  |  |  |  | |  |  |  | X |  | | X | X | X |
| Randomization to study arm | |  | X |  |  |  |  | |  |  |  |  |  | |  |  |  |
| Motivational-Interviewing based medical adherence counseling | |  | X |  |  |  | X^f^ | |  |  |  |  |  | |  |  |  |
| Motivational-Interviewing based risk reduction counseling | |  | X |  |  |  |  | |  |  |  |  | X | | X |  |  |
| Receive SVR results | |  |  |  |  |  | X | |  |  |  |  | X | |  |  |  |
| Study drug dispensation (unobserved dosing) or directly observed dosing (mDOT) ^f, g^ | |  | X | X | X | X | X | | X | X | X |  |  | |  |  |  |
| Study drug dosing compliance ^h, i^ | |  |  | X | X | X | X | | X | X | X |  |  | |  |  |  |
| Qualitative Interview | |  |  |  |  |  |  | |  |  |  | X |  | |  |  |  |
| ACASI | |  | X |  |  |  | X | |  |  |  | X |  | | X | X |  |
|  | Demographics |  | X |  |  |  |  | |  |  |  |  |  | |  |  |  |
|  | Drug use history |  | X |  |  |  |  | |  |  |  |  |  | |  |  |  |
|  | Injection drug use, injection risk behaviors |  | X |  |  |  | X | |  |  |  | X |  | | X | X |  |
|  | Quality of Life Scale (EQ5-D) |  | X |  |  |  | X | |  |  |  | X |  | | X | X |  |
|  | Depression rating scale |  | X |  |  |  | X | |  |  |  | X |  | | X | X |  |
|  | Severity of dependence scale |  | X |  |  |  | X | |  |  |  | X |  | | X | X |  |
|  | # / HCV status of drug using and injection partners |  | X |  |  |  | X | |  |  |  | X |  | | X | X |  |
|  | Partner-by-partner data on injection/substance use/HCV status |  | X |  |  |  | X | |  |  |  | X |  | | X | X |  |
|  | Participant satisfaction with treatment |  |  |  |  |  |  | |  |  |  | X |  | |  |  |  |

a The screening window can be extended with sponsor approval to 42 days in exceptional circumstances, including the need for additional HCV sequence analysis.

b Should include information regarding (a) the subject’s acquisition of HCV infection and (b) describe any past treatment for their HCV infection.

c Vital signs include resting blood pressure, pulse, respiratory rate and temperature.

d For females of childbearing potential only

e HCV genotype will either be conducted during screening or documentation of prior results will be accepted if they are confirmed to be from the same period of time during which chronic HCV was confirmed with sequential RNA results.

f May move to week 3 depending on when results are received.

g Subjects must be instructed to bring back all bottles of study drug in the original container at every post enrollment study visits through the end of treatment.

h Study drug will be reconciled at every post-enrollment visit by study staff in order to monitor the subject’s adherence with the dosing regimen.

i Enrollment assessments must be performed prior to dosing.

* There are two mandatory screening visits; activities that must be conducted in screening visit one are marked with an *.

Appendix 2. Estimating Severity Grade for Parameters Not Identified in the Grading Table

The functional table below should be used to grade the severity of an AE that is not specifically identified in the grading table. In addition, all deaths related to an AE are to be classified as grade 5.

| **PARAMETER** | **GRADE 1 MILD** | **GRADE 2 MODERATE** | **GRADE 3 SEVERE** | **GRADE 4 POTENTIALLY**  **LIFE-THREATENING** |
| --- | --- | --- | --- | --- |
| **Clinical** adverse event **NOT** identified elsewhere in the grading table | Mild symptoms causing no or minimal interference with usual social & functional activities with intervention not indicated | Moderate symptoms causing greater than minimal interference with usual social & functional activities with intervention indicated | Severe symptoms causing inability to perform usual social & functional activities with intervention or hospitalization indicated | Potentially life-threatening symptoms causing inability to perform basic self-care functions with intervention indicated to prevent permanent impairment, persistent disability, or death |

*Major Clinical Conditions*

Cardiovascular

|  |  |  |  |  |
| --- | --- | --- | --- | --- |
| **PARAMETER** | **GRADE 1 MILD** | **GRADE 2 MODERATE** | **GRADE 3 SEVERE** | **GRADE 4 POTENTIALLY LIFE- THREATENING** |
| **Arrhythmia**  (by ECG or physical examination)  *Specify type, if applicable* | No symptoms AND No intervention indicated | No symptoms AND Non-urgent intervention indicated | Non-life-threatening symptoms AND Non-urgent  intervention indicated | Life-threatening arrhythmia OR Urgent intervention indicated |
| **Blood Pressure Abnormalities**[**1**](#bookmark5)  ***Hypertension*** *(with the lowest reading taken after repeat testing during a visit)*  *≥ 18 years of age* | 140 to < 160 mmHg systolic  OR  90 to < 100 mmHg diastolic | ≥ 160 to < 180 mmHg systolic OR  ≥ 100 to < 110 mmHg diastolic | ≥ 180 mmHg systolic OR  ≥ 110 mmHg diastolic | Life-threatening consequences in a participant not previously diagnosed with hypertension (e.g., malignant hypertension) OR Hospitalization indicated |
| *< 18 years of age* | > 120/80 mmHg | ≥ 95th to < 99th percentile + 5 mmHg adjusted for age, height, and gender (systolic and/or diastolic) | ≥ 99th percentile  + 5 mmHg adjusted for age, height, and gender (systolic and/or diastolic) | Life-threatening consequences in a participant not previously diagnosed with hypertension (e.g., malignant hypertension) OR Hospitalization indicated |
| ***Hypotension*** | No symptoms | Symptoms corrected with oral fluid replacement | Symptoms AND IV fluids indicated | Shock requiring use of vasopressors or mechanical assistance to maintain blood pressure |
| **Cardiac Ischemia or Infarction**  *Report only one* | NA | NA | New symptoms with ischemia (stable angina) OR New testing consistent with ischemia | Unstable angina OR Acute myocardial infarction |
| **Heart Failure** | No symptoms AND Laboratory or cardiac imaging abnormalities | Symptoms with mild to moderate activity or exertion | Symptoms at rest or with minimal activity or exertion (e.g., hypoxemia) OR Intervention indicated (e.g., oxygen) | Life-threatening consequences OR Urgent intervention indicated (e.g., vasoactive medications, ventricular assist device, heart transplant) |
| **Hemorrhage**  (with significant acute blood loss) | NA | Symptoms AND No transfusion indicated | Symptoms AND Transfusion of ≤ 2 units packed RBCs indicated | Life-threatening hypotension OR Transfusion of > 2 units packed RBCs (for children, packed RBCs  > 10 cc/kg) indicated |
|  |  |  |  |  |

1 Blood pressure norms for children < 18 years of age can be found in: Expert Panel on Integrated Guidelines for Cardiovascular Health and Risk Reduction in Children and Adolescents. *Pediatrics* 2011;128;S213; originally published online November 14, 2011; DOI: 10.1542/peds.2009- 2107C.

Cardiovascular

|  |  |  |  |  |
| --- | --- | --- | --- | --- |
| **PARAMETER** | **GRADE 1 MILD** | **GRADE 2 MODERATE** | **GRADE 3 SEVERE** | **GRADE 4 POTENTIALLY LIFE- THREATENING** |
| **Prolonged PR Interval or AV Block**  *Report only one*  *> 16 years of age* | PR interval 0.21 to <  0.25 seconds | PR interval ≥ 0.25 seconds OR Type I 2nd degree AV block | Type II 2nd degree AV block OR Ventricular pause ≥ 3.0 seconds | Complete AV block |
| *≤ 16 years of age* | 1st degree AV block (PR interval  > normal for age and rate) | Type I 2nd degree AV block | Type II 2nd degree AV block OR Ventricular pause ≥ 3.0 seconds | Complete AV block |
| **Prolonged QTc Interval**[**2**](#bookmark6) | 0.45 to 0.47 seconds | > 0.47 to 0.50  seconds | > 0.50 seconds OR  ≥ 0.06 seconds above baseline | Life-threatening consequences (e.g., Torsade de pointes, other associated serious ventricular dysrhythmia) |
| **Thrombosis or Embolism**  *Report only one* | NA | Symptoms AND No intervention indicated | Symptoms AND Intervention indicated | Life-threatening embolic event (e.g., pulmonary embolism, thrombus) |

2 As per Bazett’s formula.

Dermatologic

|  |  |  |  |  |
| --- | --- | --- | --- | --- |
| **PARAMETER** | **GRADE 1 MILD** | **GRADE 2 MODERATE** | **GRADE 3 SEVERE** | **GRADE 4 POTENTIALLY LIFE- THREATENING** |
| **Alopecia** (scalp only) | Detectable by study participant, caregiver, or physician AND Causing no or minimal interference with usual social & functional activities | Obvious on visual inspection AND Causing greater than minimal interference with usual social & functional activities | NA | NA |
| **Bruising** | Localized to one area | Localized to more than one area | Generalized | NA |
| **Cellulitis** | NA | Non-parenteral treatment indicated (e.g., oral antibiotics, antifungals, antivirals) | IV treatment indicated (e.g., IV antibiotics, antifungals, antivirals) | Life-threatening consequences (e.g., sepsis, tissue necrosis) |
| **Hyperpigmentation** | Slight or localized causing no or minimal interference with usual social & functional activities | Marked or generalized causing greater than minimal interference with usual social & functional activities | NA | NA |
| **Hypopigmentation** | Slight or localized causing no or minimal interference with usual social & functional activities | Marked or generalized causing greater than minimal interference with usual social & functional activities | NA | NA |
| **Petechiae** | Localized to one area | Localized to more than one area | Generalized | NA |
| **Pruritus**[**3**](#bookmark8)  (without skin lesions) | Itching causing no or minimal interference with usual social & functional activities | Itching causing greater than minimal interference with usual social & functional activities | Itching causing inability to perform usual social & functional activities | NA |
| **Rash**  *Specify type, if applicable* | Localized rash | Diffuse rash OR Target lesions | Diffuse rash AND Vesicles or limited number of bullae or superficial ulcerations of mucous membrane limited to one site | Extensive or generalized bullous lesions OR Ulceration of mucous membrane involving two or more distinct mucosal sites OR Stevens- Johnson syndrome OR Toxic epidermal necrolysis |

3 For pruritus associated with injections or infusions, see the *Site Reactions to Injections and Infusions* section (page 23).

|  |  |  |  |  |
| --- | --- | --- | --- | --- |

Endocrine and Metabolic

|  |  |  |  |  |
| --- | --- | --- | --- | --- |
| **PARAMETER** | **GRADE 1 MILD** | **GRADE 2 MODERATE** | **GRADE 3 SEVERE** | **GRADE 4 POTENTIALLY LIFE- THREATENING** |
| **Diabetes Mellitus** | Controlled without medication | Controlled with medication OR Modification of current medication regimen | Uncontrolled despite treatment modification OR Hospitalization for immediate glucose control indicated | Life-threatening consequences (e.g., ketoacidosis, hyperosmolar non- ketotic coma, end organ failure) |
| **Gynecomastia** | Detectable by study participant, caregiver, or physician AND Causing no or minimal interference with usual social & functional activities | Obvious on visual inspection AND Causing pain with greater than minimal interference with usual social & functional activities | Disfiguring changes AND Symptoms requiring intervention or causing inability to perform usual social  & functional activities | NA |
| **Hyperthyroidism** | No symptoms AND Abnormal laboratory value | Symptoms causing greater than minimal interference with usual social & functional activities OR Thyroid suppression therapy indicated | Symptoms causing inability to perform usual social & functional activities OR Uncontrolled despite treatment modification | Life-threatening consequences (e.g., thyroid storm) |
| **Hypothyroidism** | No symptoms AND Abnormal laboratory value | Symptoms causing greater than minimal interference with usual social & functional activities OR Thyroid replacement therapy indicated | Symptoms causing inability to perform usual social & functional activities OR Uncontrolled despite treatment modification | Life-threatening consequences (e.g., myxedema coma) |
| **Lipoatrophy**[**4**](#bookmark10) | Detectable by study participant, caregiver, or physician AND Causing no or minimal interference with usual social & functional activities | Obvious on visual inspection AND Causing greater than minimal interference with usual social & functional activities | Disfiguring changes | NA |
| **Lipohypertrophy**[**5**](#bookmark11) | Detectable by study participant, caregiver, or physician AND Causing no or minimal interference with usual social & functional activities | Obvious on visual inspection AND Causing greater than minimal interference with usual social & functional activities | Disfiguring changes | NA |
|  |  |  |  |  |

4 Definition: A disorder characterized by fat loss in the face, extremities, and buttocks.

5 Definition: A disorder characterized by abnormal fat accumulation on the back of the neck, breasts, and abdomen.

Gastrointestinal

|  |  |  |  |  |
| --- | --- | --- | --- | --- |
| **PARAMETER** | **GRADE 1 MILD** | **GRADE 2 MODERATE** | **GRADE 3 SEVERE** | **GRADE 4 POTENTIALLY LIFE- THREATENING** |
| **Anorexia** | Loss of appetite without decreased oral intake | Loss of appetite associated with decreased oral intake without significant weight loss | Loss of appetite associated with significant weight loss | Life-threatening consequences OR Aggressive intervention indicated (e.g., tube feeding, total parenteral nutrition) |
| **Ascites** | No symptoms | Symptoms AND Intervention indicated (e.g., diuretics, therapeutic paracentesis) | Symptoms recur or persist despite intervention | Life-threatening consequences |
| **Bloating or Distension**  *Report only one* | Symptoms causing no or minimal interference with usual social & functional activities | Symptoms causing greater than minimal interference with usual social & functional activities | Symptoms causing inability to perform usual social & functional activities | NA |
| **Cholecystitis** | NA | Symptoms AND Medical intervention indicated | Radiologic, endoscopic, or operative intervention indicated | Life-threatening consequences (e.g., sepsis, perforation) |
| **Constipation** | NA | Persistent constipation requiring regular use of dietary modifications, laxatives, or enemas | Obstipation with manual evacuation indicated | Life-threatening consequences (e.g., obstruction) |
| **Diarrhea**  *≥ 1 year of age* | Transient or intermittent episodes of unformed stools OR Increase of ≤ 3 stools over baseline per 24-hour period | Persistent episodes of unformed to watery stools OR Increase of 4 to 6 stools over baseline per 24-hour period | Increase of ≥ 7 stools per 24-hour period OR IV fluid replacement indicated | Life-threatening consequences (e.g., hypotensive shock) |
| *< 1 year of age* | Liquid stools (more unformed than usual) but usual number of stools | Liquid stools with increased number of stools OR Mild dehydration | Liquid stools with moderate dehydration | Life-threatening consequences (e.g., liquid stools resulting in severe dehydration, hypotensive shock) |
| **Dysphagia or Odynophagia**  *Report only one and specify location* | Symptoms but able to eat usual diet | Symptoms causing altered dietary intake with no intervention indicated | Symptoms causing severely altered dietary intake with intervention indicated | Life-threatening reduction in oral intake |
| **Gastrointestinal Bleeding** | Not requiring intervention other than iron supplement | Endoscopic intervention indicated | Transfusion indicated | Life-threatening consequences (e.g., hypotensive shock) |

Gastrointestinal

|  |  |  |  |  |
| --- | --- | --- | --- | --- |
| **PARAMETER** | **GRADE 1 MILD** | **GRADE 2 MODERATE** | **GRADE 3 SEVERE** | **GRADE 4 POTENTIALLY LIFE- THREATENING** |
| **Mucositis or Stomatitis**  *Report only one and specify location* | Mucosal erythema | Patchy pseudomembranes or ulcerations | Confluent pseudomembranes or ulcerations OR Mucosal bleeding with minor trauma | Life-threatening consequences (e.g., aspiration, choking) OR Tissue necrosis OR Diffuse spontaneous mucosal bleeding |
| **Nausea** | Transient (< 24 hours) or intermittent AND No or minimal interference with oral intake | Persistent nausea resulting in decreased oral intake for 24 to 48 hours | Persistent nausea resulting in minimal oral intake for > 48 hours OR Rehydration indicated (e.g., IV fluids) | Life-threatening consequences (e.g., hypotensive shock) |
| **Pancreatitis** | NA | Symptoms with hospitalization not indicated | Symptoms with hospitalization indicated | Life-threatening consequences (e.g., circulatory failure, hemorrhage, sepsis) |
| **Perforation**  (colon or rectum) | NA | NA | Intervention indicated | Life-threatening consequences |
| **Proctitis** | Rectal discomfort with no intervention indicated | Symptoms causing greater than minimal interference with usual social & functional activities OR Medical intervention indicated | Symptoms causing inability to perform usual social & functional activities OR Operative intervention indicated | Life-threatening consequences (e.g., perforation) |
| **Rectal Discharge** | Visible discharge | Discharge requiring the use of pads | NA | NA |
| **Vomiting** | Transient or intermittent AND No or minimal interference with oral intake | Frequent episodes with no or mild dehydration | Persistent vomiting resulting in orthostatic hypotension OR Aggressive rehydration indicated (e.g., IV fluids) | Life-threatening consequences (e.g., hypotensive shock) |

Musculoskeletal

|  |  |  |  |  |
| --- | --- | --- | --- | --- |
| **PARAMETER** | **GRADE 1 MILD** | **GRADE 2 MODERATE** | **GRADE 3 SEVERE** | **GRADE 4 POTENTIALLY LIFE- THREATENING** |
| **Arthralgia** | Joint pain causing no or minimal interference with usual social & functional activities | Joint pain causing greater than minimal interference with usual social & functional activities | Joint pain causing inability to perform usual social & functional activities | Disabling joint pain causing inability to perform basic self-care functions |
| **Arthritis** | Stiffness or joint swelling causing no or minimal interference with usual social & functional activities | Stiffness or joint swelling causing greater than minimal interference with usual social & functional activities | Stiffness or joint swelling causing inability to perform usual social & functional activities | Disabling joint stiffness or swelling causing inability to perform basic self-care functions |
| **Myalgia** (generalized) | Muscle pain causing no or minimal interference with usual social & functional activities | Muscle pain causing greater than minimal interference with usual social & functional activities | Muscle pain causing inability to perform usual social & functional activities | Disabling muscle pain causing inability to perform basic self-care functions |
| **Osteonecrosis** | NA | No symptoms but with radiographic findings AND No operative intervention indicated | Bone pain with radiographic findings OR Operative intervention indicated | Disabling bone pain with radiographic findings causing inability to perform basic self-care functions |
| **Osteopenia**[**6**](#bookmark14)  *≥ 30 years of age* | BMD t-score  -2.5 to -1 | NA | NA | NA |
| *< 30 years of age* | BMD z-score  -2 to -1 | NA | NA | NA |
| **Osteoporosis6**  *≥ 30 years of age* | NA | BMD t-score < -2.5 | Pathologic fracture (e.g., compression fracture causing loss of vertebral height) | Pathologic fracture causing life-threatening consequences |
| *< 30 years of age* | NA | BMD z-score < -2 | Pathologic fracture (e.g., compression fracture causing loss of vertebral height) | Pathologic fracture causing life-threatening consequences |

6 BMD t and z scores can be found in: Kanis JA on behalf of the World Health Organization Scientific Group (2007). Assessment of osteoporosis at the primary health-care level. Technical Report. World Health Organization Collaborating Centre for Metabolic Bone Diseases, University of Sheffield, UK. 2007: Printed by the University of Sheffield.

Neurologic

|  |  |  |  |  |
| --- | --- | --- | --- | --- |
| **PARAMETER** | **GRADE 1 MILD** | **GRADE 2 MODERATE** | **GRADE 3 SEVERE** | **GRADE 4 POTENTIALLY LIFE- THREATENING** |
| **Acute CNS Ischemia** | NA | NA | Transient ischemic attack | Cerebral vascular accident (e.g., stroke with neurological deficit) |
| **Altered Mental Status** (for Dementia, see *Cognitive, Behavioral, or Attentional Disturbance* below) | Changes causing no or minimal interference with usual social & functional activities | Mild lethargy or somnolence causing greater than minimal interference with usual social & functional activities | Confusion, memory impairment, lethargy, or somnolence causing inability to perform usual social  & functional activities | Delirium OR Obtundation OR Coma |
| **Ataxia** | Symptoms causing no or minimal interference with usual social & functional activities OR No symptoms with ataxia detected on examination | Symptoms causing greater than minimal interference with usual social & functional activities | Symptoms causing inability to perform usual social & functional activities | Disabling symptoms causing inability to perform basic self-care functions |
| **Cognitive, Behavioral, or Attentional Disturbance** (includes dementia and attention deficit disorder)  *Specify type, if applicable* | Disability causing no or minimal interference with usual social & functional activities OR Specialized resources not indicated | Disability causing greater than minimal interference with usual social & functional activities OR Specialized resources on part- time basis indicated | Disability causing inability to perform usual social & functional activities OR Specialized resources on a full- time basis indicated | Disability causing inability to perform basic self-care functions OR Institutionalization indicated |
| **Developmental Delay**  *< 18 years of age Specify type, if applicable* | Mild developmental delay, either motor or cognitive, as determined by comparison with a developmental screening tool appropriate for the setting | Moderate developmental delay, either motor or cognitive, as determined by comparison with a developmental screening tool appropriate for the setting | Severe developmental delay, either motor or cognitive, as determined by comparison with a developmental screening tool appropriate for the setting | Developmental regression, either motor or cognitive, as determined by comparison with a developmental screening tool appropriate for the setting |
| **Headache** | Symptoms causing no or minimal interference with usual social & functional activities | Symptoms causing greater than minimal interference with usual social & functional activities | Symptoms causing inability to perform usual social & functional activities | Symptoms causing inability to perform basic self-care functions OR Hospitalization indicated OR Headache with significant impairment of alertness or other neurologic function |

Neurologic

|  |  |  |  |  |
| --- | --- | --- | --- | --- |
| **PARAMETER** | **GRADE 1 MILD** | **GRADE 2 MODERATE** | **GRADE 3 SEVERE** | **GRADE 4 POTENTIALLY LIFE- THREATENING** |
| **Neuromuscular Weakness** (includes myopathy and neuropathy)  *Specify type, if applicable* | Minimal muscle weakness causing no or minimal interference with usual social & functional activities OR No symptoms with decreased strength on examination | Muscle weakness causing greater than minimal interference with usual social & functional activities | Muscle weakness causing inability to perform usual social  & functional activities | Disabling muscle weakness causing inability to perform basic self-care functions OR Respiratory muscle weakness impairing ventilation |
| **Neurosensory Alteration** (includes paresthesia and painful neuropathy)  *Specify type, if applicable* | Minimal paresthesia causing no or minimal interference with usual social & functional activities OR No symptoms with sensory alteration on examination | Sensory alteration or paresthesia causing greater than minimal interference with usual social & functional activities | Sensory alteration or paresthesia causing inability to perform usual social & functional activities | Disabling sensory alteration or paresthesia causing inability to perform basic self-care functions |
| **Seizures**  ***New Onset Seizure***  *≥ 18 years of age* | NA | NA | 1 to 3 seizures | Prolonged and repetitive seizures (e.g., status epilepticus) OR Difficult to control (e.g., refractory epilepsy) |
| *< 18 years of age (includes new or pre- existing febrile seizures)* | Seizure lasting < 5 minutes with < 24 hours postictal state | Seizure lasting 5 to < 20 minutes with  < 24 hours postictal state | Seizure lasting ≥ 20 minutes OR > 24 hours postictal state | Prolonged and repetitive seizures (e.g., status epilepticus) OR Difficult to control (e.g., refractory epilepsy) |
| ***Pre-existing Seizure*** | NA | Increased frequency from previous level of control without change in seizure character | Change in seizure character either in duration or quality (e.g., severity or focality) | Prolonged and repetitive seizures (e.g., status epilepticus) OR Difficult to control (e.g., refractory epilepsy) |
| **Syncope** | Near syncope without loss of consciousness (e.g., pre-syncope) | Loss of consciousness with no intervention indicated | Loss of consciousness AND Hospitalization or intervention required | NA |

Pregnancy, Puerperium, and Perinatal

|  |  |  |  |  |
| --- | --- | --- | --- | --- |
| **PARAMETER** | **GRADE 1 MILD** | **GRADE 2 MODERATE** | **GRADE 3 SEVERE** | **GRADE 4 POTENTIALLY LIFE- THREATENING** |
| **Fetal Death or Stillbirth** (report using mother’s participant ID)  *Report only one* | NA | NA | Fetal loss occurring at  ≥ 20 weeks gestation | NA |
| **Preterm Delivery**[**7**](#bookmark17) (report using mother’s participant ID) | Delivery at 34 to  < 37 weeks gestational age | Delivery at 28 to  < 34 weeks gestational age | Delivery at 24 to  < 28 weeks gestational age | Delivery at < 24 weeks gestational age |
| **Spontaneous Abortion or Miscarriage**[**8**](#bookmark18) (report using mother’s participant ID)  *Report only one* | Chemical pregnancy | Uncomplicated spontaneous abortion or miscarriage | Complicated spontaneous abortion or miscarriage | NA |

7 Definition: A delivery of a live-born neonate occurring at ≥ 20 to < 37 weeks gestational age.

8 Definition: A clinically recognized pregnancy occurring at < 20 weeks gestational age.

Psychiatric

|  |  |  |  |  |
| --- | --- | --- | --- | --- |
| **PARAMETER** | **GRADE 1 MILD** | **GRADE 2 MODERATE** | **GRADE 3 SEVERE** | **GRADE 4 POTENTIALLY LIFE- THREATENING** |
| **Insomnia** | Mild difficulty falling asleep, staying asleep, or waking up early | Moderate difficulty falling asleep, staying asleep, or waking up early | Severe difficulty falling asleep, staying asleep, or waking up early | NA |
| **Psychiatric Disorders** (includes anxiety, depression, mania, and psychosis)  *Specify disorder* | Symptoms with intervention not indicated OR Behavior causing no or minimal interference with usual social & functional activities | Symptoms with intervention indicated OR Behavior causing greater than minimal interference with usual social & functional activities | Symptoms with hospitalization indicated OR Behavior causing inability to perform usual social & functional activities | Threatens harm to self or others OR Acute psychosis OR Behavior causing inability to perform basic self-care functions |
| **Suicidal Ideation or Attempt**  *Report only one* | Preoccupied with thoughts of death AND No wish to kill oneself | Preoccupied with thoughts of death AND Wish to kill oneself with no specific plan or intent | Thoughts of killing oneself with partial or complete plans but no attempt to do so OR Hospitalization indicated | Suicide attempted |

Respiratory

|  |  |  |  |  |
| --- | --- | --- | --- | --- |
| **PARAMETER** | **GRADE 1 MILD** | **GRADE 2 MODERATE** | **GRADE 3 SEVERE** | **GRADE 4 POTENTIALLY LIFE- THREATENING** |
| **Acute Bronchospasm** | Forced expiratory volume in 1 second or peak flow reduced to  ≥ 70 to < 80% OR  Mild symptoms with intervention not indicated | Forced expiratory volume in 1 second or peak flow 50 to  < 70% OR Symptoms with intervention indicated OR Symptoms causing greater than minimal interference with usual social & functional activities | Forced expiratory volume in 1 second or peak flow 25 to  < 50% OR Symptoms causing inability to perform usual social  & functional activities | Forced expiratory volume in 1 second or peak flow < 25% OR Life-threatening respiratory or hemodynamic compromise OR Intubation |
| **Dyspnea or Respiratory Distress**  *Report only one* | Dyspnea on exertion with no or minimal interference with usual social & functional activities OR Wheezing OR Minimal increase in respiratory rate for age | Dyspnea on exertion causing greater than minimal interference with usual social & functional activities OR Nasal flaring OR Intercostal retractions OR Pulse oximetry 90 to < 95% | Dyspnea at rest causing inability to perform usual social  & functional activities OR Pulse oximetry < 90% | Respiratory failure with ventilator support indicated (e.g., CPAP, BPAP, intubation) |

Sensory

|  |  |  |  |  |
| --- | --- | --- | --- | --- |
| **PARAMETER** | **GRADE 1 MILD** | **GRADE 2 MODERATE** | **GRADE 3 SEVERE** | **GRADE 4 POTENTIALLY LIFE- THREATENING** |
| **Hearing Loss**  *≥ 12 years of age* | NA | Hearing aid or intervention not indicated | Hearing aid or intervention indicated | Profound bilateral hearing loss (> 80 dB at 2 kHz and above) OR Non-serviceable hearing (i.e., >50 dB audiogram and <50% speech discrimination) |
| *< 12 years of age (based on a 1, 2, 3, 4, 6 and 8 kHz audiogram)* | > 20 dB hearing loss at ≤ 4 kHz | > 20 dB hearing loss at > 4 kHz | > 20 dB hearing loss at ≥ 3 kHz in one ear with additional speech language related services indicated (where available) OR Hearing loss sufficient to indicate therapeutic intervention, including hearing aids | Audiologic indication for cochlear implant and additional speech- language related services indicated (where available) |
| **Tinnitus** | Symptoms causing no or minimal interference with usual social & functional activities with intervention not indicated | Symptoms causing greater than minimal interference with usual social & functional activities with intervention indicated | Symptoms causing inability to perform usual social & functional activities | NA |
| **Uveitis** | No symptoms AND Detectable on examination | Anterior uveitis with symptoms OR Medicamylasal intervention indicated | Posterior or pan- uveitis OR Operative intervention indicated | Disabling visual loss in affected eye(s) |
| **Vertigo** | Vertigo causing no or minimal interference with usual social & functional activities | Vertigo causing greater than minimal interference with usual social & functional activities | Vertigo causing inability to perform usual social & functional activities | Disabling vertigo causing inability to perform basic self-care functions |
| **Visual Changes**  (assessed from baseline) | Visual changes causing no or minimal interference with usual social & functional activities | Visual changes causing greater than minimal interference with usual social & functional activities | Visual changes causing inability to perform usual social  & functional activities | Disabling visual loss in affected eye(s) |

Systemic

|  |  |  |  |  |
| --- | --- | --- | --- | --- |
| **PARAMETER** | **GRADE 1 MILD** | **GRADE 2 MODERATE** | **GRADE 3 SEVERE** | **GRADE 4 POTENTIALLY LIFE- THREATENING** |
| **Acute Allergic Reaction** | Localized urticaria (wheals) with no medical intervention indicated | Localized urticaria with intervention indicated OR Mild angioedema with no intervention indicated | Generalized urticaria OR Angioedema with intervention indicated OR Symptoms of mild bronchospasm | Acute anaphylaxis OR Life-threatening bronchospasm OR Laryngeal edema |
| **Chills** | Symptoms causing no or minimal interference with usual social & functional activities | Symptoms causing greater than minimal interference with usual social & functional activities | Symptoms causing inability to perform usual social & functional activities | NA |
| **Cytokine Release Syndrome**[**9**](#bookmark23) | Mild signs and symptoms AND Therapy (i.e., antibody infusion) interruption not indicated | Therapy (i.e., antibody infusion) interruption indicated AND Responds promptly to symptomatic treatment OR Prophylactic medications indicated for ≤ 24 hours | Prolonged severe signs and symptoms OR Recurrence of symptoms following initial improvement | Life-threatening consequences (e.g., requiring pressor or ventilator support) |
| **Fatigue or Malaise**  *Report only one* | Symptoms causing no or minimal interference with usual social & functional activities | Symptoms causing greater than minimal interference with usual social & functional activities | Symptoms causing inability to perform usual social & functional activities | Incapacitating symptoms of fatigue or malaise causing inability to perform basic self-care functions |
| **Fever** (non-axillary temperatures only) | 38.0 to < 38.6°C or 100.4 to < 101.5°F | ≥ 38.6 to < 39.3°C  or ≥ 101.5 to  < 102.7°F | ≥ 39.3 to < 40.0°C or  ≥ 102.7 to < 104.0°F | ≥ 40.0°C or ≥ 104.0°F |
| **Pain**[10](#bookmark24) (not associated with study agent injections and not specified elsewhere)  *Specify location* | Pain causing no or minimal interference with usual social & functional activities | Pain causing greater than minimal interference with usual social & functional activities | Pain causing inability to perform usual social  & functional activities | Disabling pain causing inability to perform basic self-care functions OR Hospitalization indicated |
| **Serum Sickness**[**11**](#bookmark25) | Mild signs and symptoms | Moderate signs and symptoms AND Intervention indicated (e.g., antihistamines) | Severe signs and symptoms AND Higher level intervention indicated (e.g., steroids or IV fluids) | Life-threatening consequences (e.g., requiring pressor or ventilator support) |
|  |  |  |  |  |

9 Definition: A disorder characterized by nausea, headache, tachycardia, hypotension, rash, and/or shortness of breath.

10 For pain associated with injections or infusions, see the *Site Reactions to Injections and Infusions* section (page 23).

11 Definition: A disorder characterized by fever, arthralgia, myalgia, skin eruptions, lymphadenopathy, marked discomfort, and/or dyspnea.

Systemic

|  |  |  |  |  |
| --- | --- | --- | --- | --- |
| **PARAMETER** | **GRADE 1 MILD** | **GRADE 2 MODERATE** | **GRADE 3 SEVERE** | **GRADE 4 POTENTIALLY LIFE- THREATENING** |
| **Underweight**[**12**](#bookmark26)  *> 5 to 19 years of age* | NA | WHO BMI z-score  < -2 to ≤ -3 | WHO BMI z-score  < -3 | WHO BMI z-score  < -3 with life-threatening consequences |
| *2 to 5 years of age* | NA | WHO Weight-for- height z-score  < -2 to ≤ -3 | WHO Weight-for- height z-score < -3 | WHO Weight-for-height z-score < -3 with life- threatening consequences |
| *< 2 years of age* | NA | WHO Weight-for- length z-score  < -2 to ≤ -3 | WHO Weight-for- length z-score < -3 | WHO Weight-for-length z-score < -3 with life- threatening consequences |
| **Weight Loss** (excludes postpartum weight loss) | NA | 5 to < 9% loss in body weight from baseline | ≥ 9 to < 20% loss in body weight from baseline | ≥ 20% loss in body weight from baseline OR Aggressive intervention indicated (e.g., tube feeding, total parenteral nutrition) |

12 WHO reference tables may be accessed by clicking the desired age range or by accessing the following URLs: <http://www.who.int/growthref/who2007_bmi_for_age/en/>for participants > 5 to 19 years of age and <http://www.who.int/childgrowth/standards/chart_catalogue/en/>for those ≤ 5 years of age.

Urinary

|  |  |  |  |  |
| --- | --- | --- | --- | --- |
| **PARAMETER** | **GRADE 1 MILD** | **GRADE 2 MODERATE** | **GRADE 3 SEVERE** | **GRADE 4 POTENTIALLY LIFE- THREATENING** |
| **Urinary Tract Obstruction** | NA | Signs or symptoms of urinary tract obstruction without hydronephrosis or renal dysfunction | Signs or symptoms of urinary tract obstruction with hydronephrosis or renal dysfunction | Obstruction causing life- threatening consequences |

Site Reactions to Injections and Infusions

|  |  |  |  |  |
| --- | --- | --- | --- | --- |
| **PARAMETER** | **GRADE 1 MILD** | **GRADE 2 MODERATE** | **GRADE 3 SEVERE** | **GRADE 4 POTENTIALLY LIFE- THREATENING** |
| **Injection Site Pain or Tenderness**  *Report only one* | Pain or tenderness causing no or minimal limitation of use of limb | Pain or tenderness causing greater than minimal limitation of use of limb | Pain or tenderness causing inability to perform usual social  & functional activities | Pain or tenderness causing inability to perform basic self-care function OR Hospitalization indicated |
| **Injection Site Erythema or Redness**[**13**](#bookmark29)  *Report only one*  *> 15 years of age* | 2.5 to < 5 cm in diameter OR 6.25 to  < 25 cm2 surface  area AND Symptoms causing no or minimal interference with usual social & functional activities | ≥ 5 to < 10 cm in diameter OR ≥ 25 to  < 100 cm2 surface  area OR Symptoms causing greater than minimal interference with usual social & functional activities | ≥ 10 cm in diameter OR ≥ 100 cm2 surface area OR Ulceration OR Secondary infection OR Phlebitis OR Sterile abscess OR Drainage OR Symptoms causing inability to perform usual social & functional activities | Potentially life- threatening consequences (e.g., abscess, exfoliative dermatitis, necrosis involving dermis or deeper tissue) |
| *≤ 15 years of age* | ≤ 2.5 cm in diameter | > 2.5 cm in diameter with < 50% surface area of the extremity segment involved (e.g., upper arm or thigh) | ≥ 50% surface area of the extremity segment involved (e.g., upper arm or thigh) OR Ulceration OR Secondary infection OR Phlebitis OR Sterile abscess OR Drainage | Potentially life- threatening consequences (e.g., abscess, exfoliative dermatitis, necrosis involving dermis or deeper tissue) |
| **Injection Site Induration or Swelling**  *Report only one*  *> 15 years of age* | Same as for **Injection Site Erythema or Redness,** *> 15 years of age* | Same as for **Injection Site Erythema or Redness,** *> 15 years of age* | Same as for **Injection Site Erythema or Redness,** *> 15 years of age* | Same as for **Injection Site Erythema or Redness,** *> 15 years of age* |
| *≤ 15 years of age* | Same as for **Injection Site Erythema or Redness,** *≤ 15 years of age* | Same as for **Injection Site Erythema or Redness,** *≤ 15 years of age* | Same as for **Injection Site Erythema or Redness,** *≤ 15 years of age* | Same as for **Injection Site Erythema or Redness,** *≤ 15 years of age* |
| **Injection Site Pruritus** | Itching localized to the injection site that is relieved spontaneously or in  < 48 hours of treatment | Itching beyond the injection site that is not generalized OR Itching localized to the injection site requiring ≥ 48 hours treatment | Generalized itching causing inability to perform usual social  & functional activities | NA |

13 Injection Site Erythema or Redness should be evaluated and graded using the greatest single diameter or measured surface area.

*Laboratory Values*

Chemistries

|  |  |  |  |  |
| --- | --- | --- | --- | --- |
| **PARAMETER** | **GRADE 1 MILD** | **GRADE 2 MODERATE** | **GRADE 3 SEVERE** | **GRADE 4 POTENTIALLY LIFE- THREATENING** |
| **Acidosis** | NA | pH ≥ 7.3 to < LLN | pH < 7.3 without life- threatening consequences | pH < 7.3 with life- threatening consequences |
| **Albumin, Low**  (g/dL; *g/L*) | 3.0 to < LLN  *30 to < LLN* | ≥ 2.0 to < 3.0  *≥ 20 to < 30* | < 2.0  *< 20* | NA |
| **Alkaline Phosphatase, High** | 1.25 to < 2.5 x ULN | 2.5 to < 5.0 x ULN | 5.0 to < 10.0 x ULN | ≥ 10.0 x ULN |
| **Alkalosis** | NA | pH > ULN to ≤ 7.5 | pH > 7.5 without life- threatening consequences | pH > 7.5 with life- threatening consequences |
| **ALT or SGPT, High**  *Report only one* | 1.25 to < 2.5 x ULN | 2.5 to < 5.0 x ULN | 5.0 to < 10.0 x ULN | ≥ 10.0 x ULN |
| **Amylase (Pancreatic) or Amylase (Total), High**  *Report only one* | 1.1 to < 1.5 x ULN | 1.5 to < 3.0 x ULN | 3.0 to < 5.0 x ULN | ≥ 5.0 x ULN |
| **AST or SGOT, High**  *Report only one* | 1.25 to < 2.5 x ULN | 2.5 to < 5.0 x ULN | 5.0 to < 10.0 x ULN | ≥ 10.0 x ULN |
| **Bicarbonate, Low**  (mEq/L; *mmol/L*) | 16.0 to < LLN  *16.0 to < LLN* | 11.0 to < 16.0  *11.0 to < 16.0* | 8.0 to < 11.0  *8.0 to < 11.0* | < 8.0  *< 8.0* |
| **Bilirubin**  ***Direct Bilirubin***[***14***](#bookmark32)***, High***  *> 28 days of age* | NA | NA | > ULN | > ULN with life- threatening consequences (e.g., signs and symptoms of liver failure) |
| *≤ 28 days of age* | ULN to ≤ 1 mg/dL | > 1 to ≤ 1.5 mg/dL | > 1.5 to ≤ 2 mg/dL | > 2 mg/dL |
| ***Total Bilirubin, High***  *> 28 days of age* | 1.1 to < 1.6 x ULN | 1.6 to < 2.6 x ULN | 2.6 to < 5.0 x ULN | ≥ 5.0 x ULN |
| *≤ 28 days of age* | See Appendix A. Total Bilirubin for Term and Preterm Neonates | See Appendix A. Total Bilirubin for Term and Preterm Neonates | See Appendix A. Total Bilirubin for Term and Preterm Neonates | See Appendix A. Total Bilirubin for Term and Preterm Neonates |
| **Calcium, High**  (mg/dL; *mmol/L*)  *≥ 7 days of age* | 10.6 to < 11.5  *2.65* to < *2.88* | 11.5 to < 12.5  *2.88* to < *3.13* | 12.5 to < 13.5  *3.13* to < *3.38* | ≥ 13.5  ≥ *3.38* |
| *< 7 days of age* | 11.5 to < 12.4  *2.88* to < *3.10* | 12.4 to < 12.9  *3.10* to < *3.23* | 12.9 to < 13.5  *3.23* to < *3.38* | ≥ 13.5  ≥ *3.38* |

14 Direct bilirubin > 1.5 mg/dL in a participant < 28 days of age should be graded as grade 2, if < 10% of the total bilirubin.

Chemistries

|  |  |  |  |  |
| --- | --- | --- | --- | --- |
| **PARAMETER** | **GRADE 1 MILD** | **GRADE 2 MODERATE** | **GRADE 3 SEVERE** | **GRADE 4 POTENTIALLY LIFE- THREATENING** |
| **Calcium (Ionized), High**  (mg/dL; *mmol/L*) | > ULN to < 6.0  *> ULN to < 1.5* | 6.0 to < 6.4  *1.5 to < 1.6* | 6.4 to < 7.2  *1.6 to < 1.8* | ≥ 7.2  *≥ 1.8* |
| **Calcium, Low**  (mg/dL; *mmol/L*)  *≥ 7 days of age* | 7.8 to < 8.4  *1.95* to < *2.10* | 7.0 to < 7.8  *1.75* to < *1.95* | 6.1 to < 7.0  *1.53* to < *1.75* | < 6.1  *< 1.53* |
| *< 7 days of age* | 6.5 to < 7.5  *1.63* to < *1.88* | 6.0 to < 6.5  *1.50* to < *1.63* | 5.50 to < 6.0  *1.38* to < *1.50* | < 5.50  *< 1.38* |
| **Calcium (Ionized), Low**  (mg/dL; *mmol/L*) | < LLN to 4.0  *< LLN to 1.0* | 3.6 to < 4.0  *0.9 to < 1.0* | 3.2 to < 3.6  *0.8 to < 0.9* | < 3.2  *< 0.8* |
| **Cardiac Troponin I, High** | NA | NA | NA | Levels consistent with myocardial infarction or unstable angina as defined by the local laboratory |
| **Creatine Kinase, High** | 3 to < 6 x ULN | 6 to < 10 x ULN | 10 to < 20 x ULN | ≥ 20 x ULN |
| **Creatinine, High** | 1.1 to 1.3 x ULN | > 1.3 to 1.8 x ULN  OR Increase of  > 0.3 mg/dL above baseline | > 1.8 to < 3.5  x ULN OR Increase of 1.5 to < 2.0 x above baseline | ≥ 3.5 x ULN OR  Increase of ≥ 2.0 x above baseline |
| **Creatinine Clearance**[**15**](#bookmark33)  **or eGFR, Low**  *Report only one* | NA | < 90 to 60 ml/min or ml/min/1.73 m2  OR  10 to < 30% decrease from baseline | < 60 to 30 ml/min or ml/min/1.73 m2  OR  ≥ 30 to < 50% decrease from baseline | < 30 ml/min or ml/min/1.73 m2 OR  ≥ 50% decrease from baseline or dialysis needed |
| **Glucose**  (mg/dL; *mmol/L*)  ***Fasting, High*** | 110 to 125  *6.11 to < 6.95* | > 125 to 250  *6.95 to < 13.89* | > 250 to 500  *13.89 to < 27.75* | > 500  ≥ *27.75* |
| ***Nonfasting, High*** | 116 to 160  *6.44 to < 8.89* | > 160 to 250  *8.89 to < 13.89* | > 250 to 500  *13.89 to < 27.75* | > 500  ≥ *27.75* |
| **Glucose, Low**  (mg/dL; *mmol/L*)  *≥ 1 month of age* | 55 to 64  *3.05 to 3.55* | 40 to < 55  *2.22 to < 3.05* | 30 to < 40  *1.67 to < 2.22* | < 30  *< 1.67* |
| *< 1 month of age* | 50 to 54  *2.78 to 3.00* | 40 to < 50  *2.22 to < 2.78* | 30 to < 40  *1.67 to < 2.22* | < 30  *< 1.67* |
| **Lactate, High** | ULN to < 2.0  x ULN without acidosis | ≥ 2.0 x ULN without acidosis | Increased lactate with pH < 7.3 without life- threatening consequences | Increased lactate with pH < 7.3 with life- threatening consequences |
|  |  |  |  |  |

15 Use the applicable formula (i.e., Cockroft-Gault in mL/min or Schwatrz in mL/min/1.73m2).

Chemistries

|  |  |  |  |  |
| --- | --- | --- | --- | --- |
| **PARAMETER** | **GRADE 1 MILD** | **GRADE 2 MODERATE** | **GRADE 3 SEVERE** | **GRADE 4 POTENTIALLY LIFE- THREATENING** |
| **Lipase, High** | 1.1 to < 1.5 x ULN | 1.5 to < 3.0 x ULN | 3.0 to < 5.0 x ULN | ≥ 5.0 x ULN |
| **Lipid Disorders**  (mg/dL; *mmol/L*)  ***Cholesterol, Fasting, High***  *≥ 18 years of age* | 200 to < 240  *5.18 to < 6.19* | 240 to < 300  *6.19 to < 7.77* | ≥ 300  ≥ *7.77* | NA |
| *< 18 years of age* | 170 to < 200  *4.40 to < 5.15* | 200 to < 300  *5.15 to < 7.77* | ≥ 300  ≥ *7.77* | NA |
| ***LDL, Fasting, High***  *≥ 18 years of age* | 130 to < 160  *3.37 to < 4.12* | 160 to < 190  *4.12 to < 4.90* | ≥ 190  *≥ 4.90* | NA |
| *> 2 to < 18 years of age* | 110 to < 130  *2.85 to < 3.34* | 130 to < 190  *3.34 to < 4.90* | ≥ 190  *≥ 4.90* | NA |
| ***Triglycerides, Fasting, High*** | 150 to 300  *1.71 to 3.42* | >300 to 500  *>3.42 to 5.7* | >500 to < 1,000  *>5.7 to 11.4* | > 1,000  *> 11.4* |
| **Magnesium**[**16**](#bookmark34)**, Low**  (mEq/L; *mmol/L*) | 1.2 to < 1.4  *0.60 to < 0.70* | 0.9 to < 1.2  *0.45 to < 0.60* | 0.6 to < 0.9  *0.30 to < 0.45* | < 0.6  *< 0.30* |
| **Phosphate, Low**  (mg/dL; *mmol/L*)  *> 14 years of age* | 2.0 to < LLN  *0.81 to < LLN* | 1.4 to < 2.0  *0.65 to < 0.81* | 1.0 to < 1.4  *0.32 to < 0.65* | < 1.0  *< 0.32* |
| *1 to 14 years of age* | 3.0 to < 3.5  *0.97 to < 1.13* | 2.5 to < 3.0  *0.81 to < 0.97* | 1.5 to < 2.5  *0.48 to < 0.81* | < 1.5  *< 0.48* |
| *< 1 year of age* | 3.5 to < 4.5  *1.13 to < 1.45* | 2.5 to < 3.5  *0.81 to < 1.13* | 1.5 to < 2.5  *0.48 to < 0.81* | < 1.5  *< 0.48* |
| **Potassium, High**  (mEq/L; *mmol/L*) | 5.6 to < 6.0  *5.6 to < 6.0* | 6.0 to < 6.5  *6.0 to < 6.5* | 6.5 to < 7.0  *6.5 to < 7.0* | ≥ 7.0  ≥ *7.0* |
| **Potassium, Low**  (mEq/L; *mmol/L*) | 3.0 to < 3.4  *3.0 to < 3.4* | 2.5 to < 3.0  *2.5 to < 3.0* | 2.0 to < 2.5  *2.0 to < 2.5* | < 2.0  *< 2.0* |
| **Sodium, High**  (mEq/L; *mmol/L*) | 146 to < 150  *146 to < 150* | 150 to < 154  *150 to < 154* | 154 to < 160  *154 to < 160* | ≥ 160  ≥ *160* |
| **Sodium, Low**  (mEq/L; *mmol/L*) | 130 to < 135  *130 to < 135* | 125 to < 130  *125 to < 135* | 121 to < 125  *121 to < 125* | ≤ 120  ≤ *120* |
| **Uric Acid, High**  (mg/dL; *mmol/L*) | 7.5 to < 10.0  *0.45 to < 0.59* | 10.0 to < 12.0  *0.59 to < 0.71* | 12.0 to < 15.0  *0.71 to < 0.89* | ≥ 15.0  ≥ *0.89* |

16 To convert a magnesium value from mg/dL to mmol/L, laboratories should multiply by 0.4114.

Hematology

|  |  |  |  |  |
| --- | --- | --- | --- | --- |
| **PARAMETER** | **GRADE 1 MILD** | **GRADE 2 MODERATE** | **GRADE 3 SEVERE** | **GRADE 4 POTENTIALLY LIFE- THREATENING** |
| **Absolute CD4+ Count,**  **Low**  (cell/mm3; *cells/L*)  *> 5 years of age (not HIV infected)* | 300 to < 400  *300 to < 400* | 200 to < 300  *200 to < 300* | 100 to < 200  *100 to < 200* | < 100  *< 100* |
| **Absolute Lymphocyte Count, Low**  (cell/mm3; *cells/L*)  *> 5 years of age (not HIV infected)* | 600 to < 650  *0.600 x 109 to*  *< 0.650 x 109* | 500 to < 600  *0.500 x 109 to*  *< 0.600 x 109* | 350 to < 500  *0.350 x 109 to*  *< 0.500 x 109* | < 350  *< 0.350 x 109* |
| **Absolute Neutrophil Count (ANC), Low**  (cells/mm3; *cells/L*)  *> 7 days of age* | 800 to 1,000  *0.800 x 109 to 1.000*  *x 109* | 600 to 799  *0.600 x 109 to 0.799 x*  *109* | 400 to 599  *0.400 x 109 to 0.599 x*  *109* | < 400  *< 0.400 x 109* |
| *2 to 7 days of age* | 1,250 to 1,500  *1.250 x 109 to 1.500*  *x 109* | 1,000 to 1,249  *1.000 x 109 to 1.249 x*  *109* | 750 to 999  *0.750 x 109 to 0.999 x*  *109* | < 750  *< 0.750 x 109* |
| *≤ 1 day of age* | 4,000 to 5,000  *4.000 x 109 to*  *5.000 x 109* | 3,000 to 3,999  *3.000 x 109 to 3.999 x*  *109* | 1,500 to 2,999  *1.500 x 109 to 2.999 x*  *109* | < 1,500  *< 1.500 x 109* |
| **Fibrinogen, Decreased**  (mg/dL; *g/L*) | 100 to < 200  *1.00 to* < *2.00*  OR  0.75 to < 1.00 x LLN | 75 to < 100  *0.75 to < 1.00*  OR  ≥ 0.50 to < 0.75 x LLN | 50 to < 75  *0.50 to < 0.75*  OR  0.25 to < 0.50 x LLN | < 50  < *0.50*  OR  < 0.25 x LLN  OR Associated with gross bleeding |
| **Hemoglobin**[**17**](#bookmark36)**, Low**  (g/dL; *mmol/L*)[18](#bookmark37)  *≥ 13 years of age (male only)* | 10.0 to 10.9  *6.19 to 6.76* | 9.0 to < 10.0  *5.57 to < 6.19* | 7.0 to < 9.0  *4.34 to < 5.57* | < 7.0  *< 4.34* |
| *≥ 13 years of age (female only)* | 9.5 to 10.4  *5.88 to 6.48* | 8.5 to < 9.5  *5.25 to < 5.88* | 6.5 to < 8.5  *4.03 to < 5.25* | < 6.5  *< 4.03* |

17 Male and female sex are defined as sex at birth.

18 The conversion factor used to convert g/dL to mmol/L is 0.6206 and is the most commonly used conversion factor. For grading hemoglobin results obtained by an analytic method with a conversion factor other than 0.6206, the result must be converted to g/dL using the appropriate conversion factor for the particular laboratory.

Hematology

|  |  |  |  |  |
| --- | --- | --- | --- | --- |
| **PARAMETER** | **GRADE 1 MILD** | **GRADE 2 MODERATE** | **GRADE 3 SEVERE** | **GRADE 4 POTENTIALLY LIFE- THREATENING** |
| *57 days of age to < 13 years of age*  *(male and female)* | 9.5 to 10.4  *5.88 to 6.48* | 8.5 to < 9.5  *5.25 to < 5.88* | 6.5 to < 8.5  *4.03 to < 5.25* | < 6.5  *< 4.03* |
| *36 to 56 days of age (male and female)* | 8.5 to 9.6  *5.26 to 5.99* | 7.0 to < 8.5  *4.32 to < 5.26* | 6.0 to < 7.0  *3.72 to < 4.32* | < 6.0  *< 3.72* |
| *22 to 35 days of age (male and female)* | 9.5 to 11.0  *5.88 to 6.86* | 8.0 to < 9.5  *4.94 to < 5.88* | 6.7 to < 8.0  *4.15 to < 4.94* | < 6.7  *< 4.15* |
| *8 to ≤ 21 days of age (male and female)* | 11.0 to 13.0  *6.81 to 8.10* | 9.0 to < 11.0  *5.57 to < 6.81* | 8.0 to < 9.0  *4.96 to < 5.57* | < 8.0  *< 4.96* |
| *≤ 7 days of age (male and female)* | 13.0 to 14.0  *8.05 to 8.72* | 10.0 to < 13.0  *6.19 to < 8.05* | 9.0 to < 10.0  *5.59 to < 6.19* | < 9.0  *< 5.59* |
| **INR, High**  (not on anticoagulation therapy) | 1.1 to < 1.5 x ULN | 1.5 to < 2.0 x ULN | 2.0 to < 3.0 x ULN | ≥ 3.0 x ULN |
| **Methemoglobin**  (% hemoglobin) | 5.0 to < 10.0% | 10.0 to < 15.0% | 15.0 to < 20.0% | ≥ 20.0% |
| **PTT, High**  (not on anticoagulation therapy) | 1.1 to < 1.66 x ULN | 1.66 to < 2.33 x ULN | 2.33 to < 3.00 x ULN | ≥ 3.00 x ULN |
| **Platelets, Decreased**  (cells/mm3; *cells/L*) | 100,000 to  < 124,999  *100.000 x 109 to*  *< 124.999 x 109* | 50,000 to  < 100,000  *50.000 x 109 to*  *< 100.000 x 109* | 25,000 to  < 50,000  *25.000 x 109 to*  *< 50.000 x 109* | < 25,000  *< 25.000 x 109* |
| **PT, High**  (not on anticoagulation therapy | 1.1 to < 1.25 x ULN | 1.25 to < 1.50 x ULN | 1.50 to < 3.00 x ULN | ≥ 3.00 x ULN |
| **WBC, Decreased**  (cells/mm3; *cells/L*)  *> 7 days of age* | 2,000 to 2,499  *2.000 x 109 to 2.499*  *x 109* | 1,500 to 1,999  *1.500 x 109 to 1.999 x*  *109* | 1,000 to 1,499  *1.000 x 109 to 1.499 x*  *109* | < 1,000  *< 1.000 x 109* |
| *≤ 7 days of age* | 5,500 to 6,999  *5.500 x 109 to 6.999*  *x 109* | 4,000 to 5,499  *4.000 x 109 to 5.499 x*  *109* | 2,500 to 3,999  *2.500 x 109 to 3.999 x*  *109* | < 2,500  *< 2.500 x 109* |

Urinalysis

|  |  |  |  |  |
| --- | --- | --- | --- | --- |
| **PARAMETER** | **GRADE 1 MILD** | **GRADE 2 MODERATE** | **GRADE 3 SEVERE** | **GRADE 4 POTENTIALLY LIFE- THREATENING** |
| **Glycosuria (**random collection tested by dipstick) | Trace to 1+ or  ≤ 250 mg | 2+ or ˃ 250 to  ≤ 500 mg | > 2+ or > 500 mg | NA |
| **Hematuria** (not to be reported based on dipstick findings or on blood believed to be of menstrual origin) | 6 to < 10 RBCs per high power field | ≥ 10 RBCs per high power field | Gross, with or without clots OR With RBC casts OR Intervention indicated | Life-threatening consequences |
| **Proteinuria** (random collection tested by dipstick) | 1+ | 2+ | 3+ or higher | NA |

Total Bilirubin Table for Term and Preterm Neonates

|  |  |  |  |  |
| --- | --- | --- | --- | --- |
| **PARAMETER** | **GRADE 1 MILD** | **GRADE 2 MODERATE** | **GRADE 3 SEVERE** | **GRADE 4 POTENTIALLY LIFE- THREATENING** |
| **Total Bilirubin**[19](#bookmark40)**, High**  (mg/dL; *µmol/L*)[20](#bookmark41)  ***Term Neonate***[***21***](#bookmark42)  *< 24 hours of age* | 4 to < 7  *68.4 to < 119.7* | 7 to < 10  *119.7 to < 171* | 10 to < 17  *171* to < *290.7* | ≥ 17  ≥ *290.7* |
| *24 to < 48 hours of age* | 5 to < 8  *85.5 to < 136.8* | 8 to < 12  *136.8 to < 205.2* | 12 to < 19  *205.2 to* < *324.9* | ≥ 19  ≥ *324.9* |
| *48 to < 72 hours of age* | 8.5 to < 13  *145.35 to < 222.3* | 13 to < 15  *222.3 to < 256.5* | 15 to < 22  *256.5 to* < *376.2* | ≥ 22  ≥ *376.2* |
| *72 hours to < 7 days of age* | 11 to < 16  *188.1 to < 273.6* | 16 to < 18  *273.6 to < 307.8* | 18 to < 24  *307.8 to* < *410.4* | ≥ 24  ≥ *410.4* |
| *7 to 28 days of age (breast feeding)* | 5 to < 10  *85.5 to < 171* | 10 to < 20  *171 to < 342* | 20 to < 25  *342 to < 427.5* | ≥ 25  ≥ *427.5* |
| *7 to 28 days of age (not breast feeding)* | 1.1 to < 1.6 x ULN | 1.6 to < 2.6 x ULN | 2.6 to < 5.0 x ULN | ≥ 5.0 x ULN |
| ***Preterm Neonate20***  *35 to < 37 weeks gestational age* | Same as for ***Total Bilirubin, High, Term Neonate*** (based on days of age). | Same as for ***Total Bilirubin, High, Term Neonate*** (based on days of age). | Same as for ***Total Bilirubin, High, Term Neonate*** (based on days of age). | Same as for ***Total Bilirubin, High, Term Neonate*** (based on days of age). |
| *32 to < 35 weeks gestational age and*  *< 7 days of age* | NA | NA | 10 to < 14  *171 to* < *239.4* | ≥ 14  *≥ 239.4* |
| *28 to < 32 weeks gestational age and*  *< 7 days of age* | NA | NA | 6 to < 10  *102.6 to < 171* | ≥ 10  *≥ 171* |
| *< 28 weeks gestational age and*  *< 7 days of age* | NA | NA | 5 to < 8  *85.5 to < 136.8* | ≥ 8  *≥ 136.8* |
| *7 to 28 days of age (breast feeding)* | 5 to < 10  *85.5 to < 171* | 10 to < 20  *171 to < 342* | 20 to < 25  *342 to < 427.5* | ≥ 25  ≥ *427.5* |
| *7 to 28 days of age (not breast feeding)* | 1.1 to < 1.6 x ULN | 1.6 to < 2.6 x ULN | 2.6 to < 5.0 x ULN | ≥ 5.0 x ULN |

19 Severity grading for total bilirubin in neonates is complex because of rapidly changing total bilirubin normal ranges in the first week of life

followed by the benign phenomenon of breast milk jaundice after the first week of life. Severity grading in this appendix corresponds approximately to cut-offs for indications for phototherapy at grade 3 and for exchange transfusion at grade 4.

20 A laboratory value of 1 mg/dL is equivalent to 17.1 µmol/L.

21 Definitions: Term is defined as ≥ 37 weeks gestational age; near-term, as ≥ 35 weeks gestational age; preterm, as < 35 weeks gestational age; and neonate, as 0 to 28 days of age.
